# Fast *drosophila* enterocyte regrowth after infection involves a reverse metabolic flux driven by an amino acid transporter

**DOI:** 10.1016/j.isci.2023.107490

**Published:** 2023-07-27

**Authors:** Catherine Socha, Inês S. Pais, Kwang-Zin Lee, Jiyong Liu, Samuel Liégeois, Matthieu Lestradet, Dominique Ferrandon

**Affiliations:** 1 Université de Strasbourg, CNRS, RIDI UPR 9022, F67084 Strasbourg, France; 2 Sino-French Hoffmann Institute, Guangzhou Medical University, Xinzao, Panyu District, Guangzhou 511436, Guangdong Province, China

**Keywords:** Natural sciences, Biological sciences, Zoology, Biochemistry, Parasitology

## Abstract

Upon exposure to a bacterial pore-forming toxin, enterocytes rapidly purge their apical cytoplasm into the gut lumen, resulting in a thin intestinal epithelium. The enterocytes regain their original shape and thickness within 16 h after the ingestion of the bacteria. Here, we show that the regrowth of *Drosophila* enterocytes entails an inversion of metabolic fluxes from the organism back toward the intestine. We identify a proton-assisted transporter, Arcus, that is required for the reverse absorption of amino acids and the timely recovery of the intestinal epithelium. Arcus is required for a peak of amino acids appearing in the hemolymph shortly after infection. The regrowth of enterocytes involves the insulin signaling pathway and Myc. The purge decreases *Myc* mRNA levels, which subsequently remain at low levels in the *arcus* mutant. Interestingly, the action of *arcus* and *Myc* in the intestinal epithelium is not cell-autonomous, suggesting amino acid fluxes within the intestinal epithelium.

## Highlights

• Purged enterocyte regrowth relies on a reverse flux of amino acids from host to gut
• A rapid upsurge in hemolymph amino acids (aa) requires the G/A/P Arcus transporter
• Basally located Arcus may act in concert with MYC-mediated enterocyte regrowth
• Clonal analysis of *Arcus* suggests a passage of aa between neighboring enterocytes

## Graphical abstract

**Figure:**
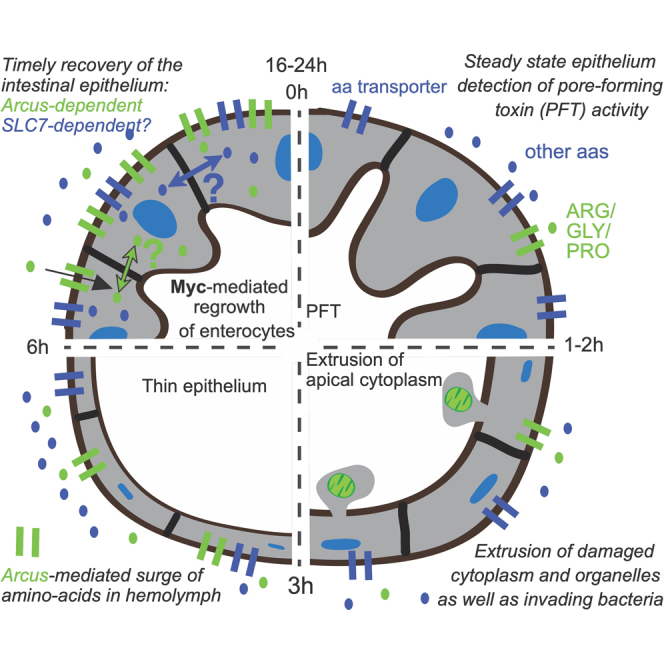

## Introduction

The intestinal tract is one of the largest interfaces between the host body and the external environment, harboring a diverse microbial community that is mostly beneficial for the host.^1^ However, the proliferation of existing opportunistic pathogens as well as the exposure to food-borne pathogens can severely compromise host health.^2^ Resistance and resilience mechanisms in the host have been selected during evolution that ensure the homeostasis of the epithelium and limit damages inflicted by pathogens.^3^ Whereas resistance mechanisms usually reduce the pathogen burden, resilience/disease tolerance mechanisms allow the host to endure damages inflicted by pathogens or by its own immune response.^4^^,^^5^ The compensatory proliferation of intestinal stem cells (ISCs) is an example of a resilience response that maintains gut homeostasis by replacing damaged gut enterocytes.^6^

Amino acids are important in determining the outcome of an infection.^7^ Amino acid metabolism affects the host physiology and may serve as an energy source. In mammals, 59 amino acid transporters have been identified and categorized in 12 different solute carrier (SLC) families such as the proton-coupled amino acid transporters (SLC36 or PATs), the sodium-coupled neutral amino acid transporters (SLC38 or SNATs), the cationic (SLC7 or CATs) and the heterodimeric (SLC7/SCL3 or HATs) amino acid transporters.^8^^,^^9^ In *Drosophila*, 603 genes are predicted to encode putative transporter proteins, which correspond to 4% of the genome.^10^ Amino acid transporters may have redundant functions, as different transporters can carry the same amino acid. These characteristics allow adaptation to various environmental conditions.

*Serratia marcescens* (*Sm*) is a Gram-negative opportunistic human pathogen that secretes several virulence factors, such as proteases and pore-forming toxins. When injected directly in the hemolymph, *Sm* resists the systemic immune response essentially because of the O-antigen of its LPS and is highly virulent.^11^^,^^12^ In contrast, *Sm* is much less virulent in an intestinal infection model. Hemocyte-mediated phagocytosis in the body cavity is an effective response against the ingested *Sm* bacteria that manage to cross the intestinal barrier. Several genes required for susceptibility or for resistance against ingested *Sm* have been identified before in a genome-wide screen.^13^

An early epithelial defense against ingested *Sm* is the cytoplasmic purge of enterocytes triggered by hemolysin produced by *Sm*Db11.^14^ Starting from 1 h after ingestion, the enterocytes of the midgut extrude a considerable portion of their cytoplasm through an aperture that is formed in the apical part of the cell. Damaged organelles such as mitochondria, and also likely toxins and invading bacteria, are actively extruded out of the exposed enterocytes. As a result of this purge, the epithelium becomes very thin and flat throughout the midgut. Here, we focus on enterocytes of the R2 region, which lose through the purge their dome-shaped apical domains typical of this portion of the midgut. The cytoplasmic purge limits damages to enterocytes as there is no increased enterocyte cell death nor compensatory stem cell proliferation during the early stages of the infection. Strikingly, the same cells are able to recover their normal shape and size within the first 20 h post infection.^14^ However, the processes required for the fast reestablishment of the epithelium remain unknown.

Here, we explore this question and show that a retrograde transport of metabolites occurs from the hemolymph to the enterocytes during the recovery of the epithelium. We found that the GLY, ALA, and PRO amino acid transporter CG1139 localizes basally to the labyrinth region of the enterocytes, a zone of intensive exchange between the enterocyte and the hemolymph, and is required for the retrograde transport of a MET analogue. A short-lived peak of amino acids in the hemolymph that occurs a few hours after challenge is dependent on this transporter. Moreover, CG1139 is required for the fast regrowth of the epithelium, but a recovery is still possible in its absence, albeit slower, suggesting a possible redundancy with other amino acid transporters shown to be potentially promoting also the regrowth of thinned enterocytes. Our data suggest that CG1139 effects may be mediated by MYC. Unexpectedly for a transmembrane protein, we found CG1139 to act non-cell autonomously and we suggest that amino acids likely circulate between wild-type and mutant enterocytes. We propose to name this transporter Arcus (ACS). Arcus is a female Roman deity homologous to the Greek Iris that personifies the rainbow and that replenishes rain clouds.

## Results

### A retrograde transport from the hemolymph to the gut takes place upon infection

**Figure 1:**
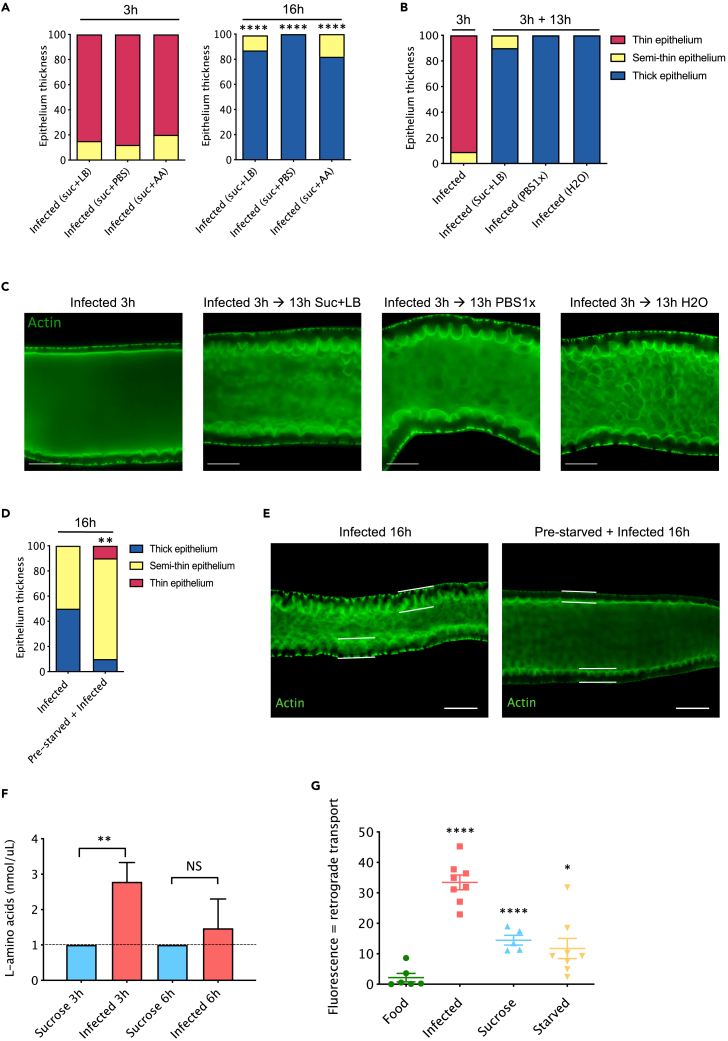
Recovery of enterocytes after infection entails retrograde transport from body reserves (A) % of epithelium thickness categories from flies infected with *Sm*Db11 resuspended in sucrose solution with 10% LB, PBS1x or a mix of essential and non-essential amino acids (chi-square, ∗∗∗∗ = p < 0.0001, comparisons between 16h and 3h from corresponding samples). Dissected midguts were qualitatively assigned after microscopic examination to either of three categories: thin epithelium (red), semi-thin epithelium (yellow) or thick epithelium (blue) (n = 24–30 per experimental condition, pooled from two to three independent experiments). (B and C) (B) % epithelium thickness categories from flies infected for 3h with *Sm*Db11 in PBS1x solution, and after transfer to sucrose with 10% LB, PBS1x or H2O (3h + 13h). For each condition, representative pictures of gut enterocytes are shown in (C). The measured epithelial thicknesses of midguts issued from an independent experiment are presented in Figure S1A. (D and E) (D) % epithelium thickness categories from flies pre-fed in standard food or pre-starved for 24h after *Sm*Db11 infection. Representative pictures of gut enterocytes are shown in (E). (F) Quantification of free amino acids in the hemolymph of flies 3h and 6h after sucrose or *Sm*Db11 infection. Bars represent the mean and the error bars display Standard Deviation of the data (one-way ANOVA, ∗∗ = p < 0.001, n = 3 × 20 hemolymphs). (G) Flies were injected with 50 μM AHA and exposed to regular food, *Sm*Db11 (infected), sucrose or water (starved). Midguts were dissected 6h later and fluorescence from the incorporated amino acid was assessed by staining with an alkyne probe and measured using ImageJ (One-way ANOVA; ∗ = p < 0.05; ∗∗∗∗ = p < 0.0001, n = 5–8). Stainings are shown in Figure S2A. The median bars represent the mean while the error bars display Standard Deviation of the data. (C, E) Green = Actin. Scale bars: 50μm (C) and 100μm (E).

Upon *Sm*Db11 ingestion, the gut epithelium becomes thin with a reduction of about two-fold in thickness.^14^ The elements required for the regrowth of enterocytes may either be derived from food or originate from the fly metabolic stores. In our usual infection solution, bacteria are mixed with 10% of lysogeny broth (LB) medium in 50mM sucrose. Removing LB from the solution did not prevent epithelial thinning at 3h nor recovery, which was also as efficient as a solution in which amino acids had been added instead of LB (Figure 1A). Next, we exposed the flies for 3 h to *Sm*Db11 re-suspended in a PBS1x solution and then placed the flies for 13 h on sterile PBS1x or to H2O, or onto sucrose with LB as a control. Most flies were able to restore a thick epithelium in the absence of any external nutrients (Figures 1B, 1C, and S1A). These results suggest that upon infection, the fast recovery of the gut epithelial cells from the cytoplasmic purge is not dependent on newly acquired nutrients, but rather on metabolites stored in the insect body.

To explore this possibility, we pre-starved flies for 24 h in H2O to deplete their internal metabolic stores. Only 10% of the flies were able to recover to a normal epithelium, while the others 80% and 10% displayed respectively a semi-thin or thin epithelium (Figures 1D and 1E). These results show that the gut epithelium cannot recover efficiently 16h post-infection (p.i.) in pre-starved flies.

Next, we directly tested a possible retrograde transport of metabolites from the rest of the body to the gut epithelium. At 3h but not 6h p.i., we observed a transient increase of free amino acids in the hemolymph of flies (Figure 1F). We injected an L-azidohomoalanine (AHA) methionine analogue in the hemocoel of flies and exposed afterward the injected flies to either standard fly food, *Sm*Db11, sucrose or to water (starved flies) for 6h. After reacting AHA with an alkyne fluorophore probe in a Click-iT reaction, the incorporated analog was detected by fluorescence in tissues in which translation is taking place.^15^ Hardly any fluorescence was measured in the gut of uninfected flies in contrast to infected ones, which displayed a large increase in midgut epithelium fluorescence 6 h p.i. Interestingly, flies that were placed on sucrose or starved on water also displayed an augmented midgut epithelium fluorescence (Figures 1G and S2A). These results show that the modified injected amino acid is being taken up from the hemolymph and incorporated into the enterocytes during the recovery phase of the cytoplasmic purge or upon amino acid starvation. Altogether, these experiments indicate the occurrence of a retrograde transport to the midgut epithelium in response to infection or starvation.

### The amino acid transporter ACS/CG1139 is required for the fast recovery from the gut epithelium after the cytoplasmic purge

**Figure 2:**
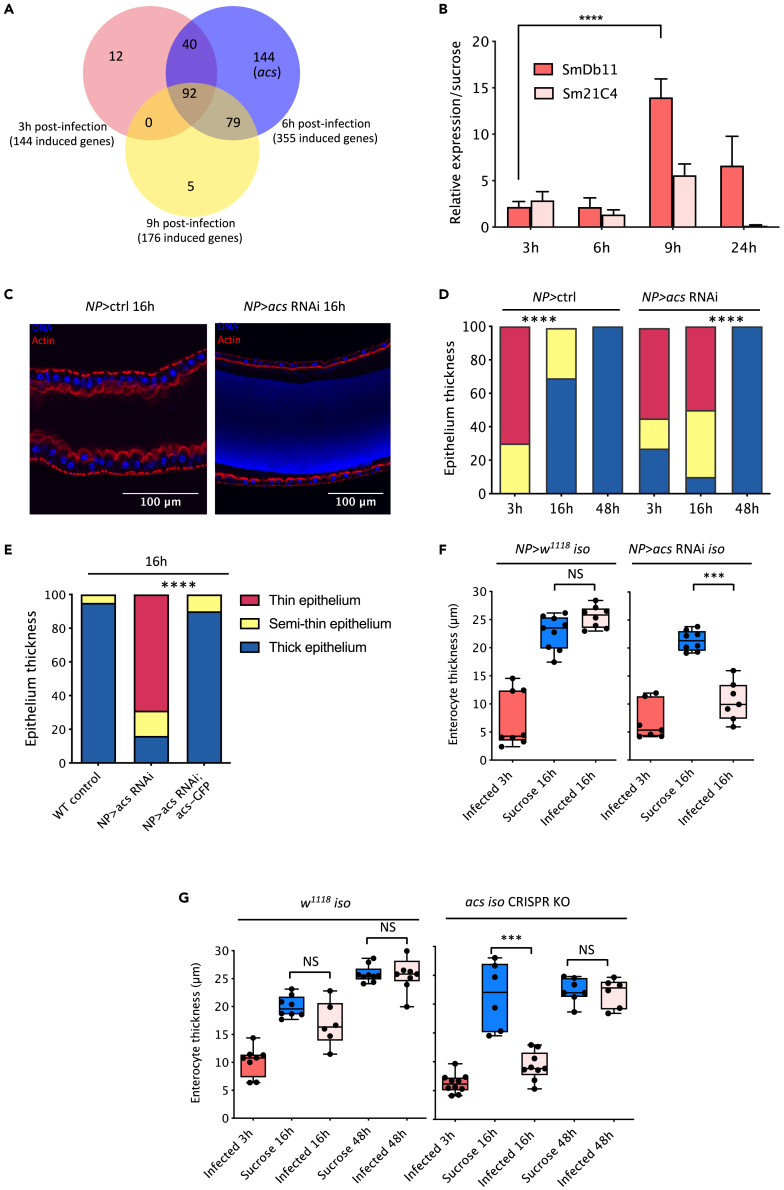
Fast recovery of gut epithelial thickness requires the ACS amino acid transporter (A) Genes induced in the midgut 3h, 6h and 9h post *Sm*Db11 infection identified by RNA-sequencing. *acs* was also induced at the 9-h time point but not significantly. (B) Gene expression analyzed by RT-qPCR of *acs* after *Sm*Db11 or *Sm*21C4 infection relative to sucrose. Bars represent the mean and the error bars display Standard Deviation of the data (one-way ANOVA, ∗∗∗∗ = p < 0,0001, n = 3 × 10 midguts). (C) Midguts from control and *NP>acs* RNAi flies 16h post *Sm*Db11 infection. (D) % epithelium thickness categories after *Sm*Db11 infection in control and *NP>acs* RNAi flies (chi-square, ∗∗∗∗ = p < 0.0001). (E) % epithelium thickness categories 16h after *Sm*Db11 infection in *NP>*ctrl, *NP>acs* RNAi and in the rescued line *NP>acs-*RNAi; UAS-*acs*-*GFP* (chi-square, ∗∗∗∗ = p < 0.0001). (F) Control and *NP > acs* RNAi *iso* flies were exposed to sucrose or *Sm*Db11 and enterocytes thickness was measured with ImageJ. Each dot represents the average of 10 enterocytes in one midgut (*lmer*, ∗∗∗ = p < 0.001, n = 7–9 midguts). (G) *w*^*1118*^*iso* and a Knock-out CRISPR mutant for *acs* (*acs iso* CRISPR KO) were exposed to sucrose or *Sm*Db11 and the thickness of the enterocytes was measured (*lmer*, ∗∗∗ = p < 0.001, n = 6–10 midguts). D-E: Qualitative quantification of intestines according to their epithelial thickness: Thin-red, Semithin-yellow, Thick-blue (n = 24–30). F-G: the middle bar of box plots represents the median and the upper and lower limits of the boxes indicate, respectively, the first and third quartile; the whiskers define the minima and maxima.

Given the results above, we next screened for altered recovery 28 putative amino acids transporter genes expressed in the *Drosophila* midgut by silencing their gene expression in enterocytes by RNA interference (RNAi) with a driver (*NP1-Gal4*, *Gal80*^*ts*^ = NP) that is mostly expressed in enterocytes (Figure S1B). Among eight hits with a strongly impaired recovery phenotype at 16h p.i., the leading one is encoded by *acs*, which remarkably was the sole transporter gene found to be up-regulated 6h p.i. in an RNA-seq experiment that was previously performed in our laboratory (Figures 2A, 2C, and 2D). We validated the RNA-seq results by RT-qPCR and observed a more than 10-fold up-regulation of *acs* transcripts at 9 h p.i., which did not occur to the same extent upon a challenge with *Sm*21C4, an *S. marcescens* mutant for hemolysin that does not cause an accentuated cytoplasmic purge^14^ (Figure 2B).

*acs* encodes an amino acid transporter similar to a mammalian PAT (Proton-assisted SLC36 Amino acid Transporter) (Figure S1C). It has previously been shown in *D. melanogaster* to promote cell growth and has been proposed to act as a transceptor involved in mTOR activation.^16^^,^^17^ The possibility that this transporter might regulate the recovery phase that follows the cytoplasmic purge prompted us to investigate further its role in enterocytes.

Having validated the efficiency of *NP>acs* RNAi (Figure S1D), we confirmed that *acs* function was indeed required in enterocytes by repeating the RNA silencing experiment with another enterocyte-specific driver, *mex1-Gal4*, and found also an impaired recovery in the *acs*-silenced midgut epithelia 16 h after *Sm* ingestion (Figure S1E). Next, we retested *NP>acs* RNAi flies 16 and 48 h p.i. Whereas the silenced flies displayed an impaired epithelial recovery as compared to control flies at 16h, they had fully recovered by 48h (Figure 2D). The 16h delayed recovery phenotype is indeed due to the silencing of the *acs* gene as it was observed with an independent RNAi line (Figure S1F) and was rescued by the ectopic expression of an *acs-GFP* transgene^17^ (Figure 2E).

To limit possible effects of the genetic background of *acs* RNAi, we isogenized this line into the *w*^*1118*^ *iso* background and measured more precisely the thickness of enterocytes. Overall, we obtained similar results to those obtained with the non-isogenized lines reported so far. In sucrose, both NP > *iso* and NP > *acs* RNAi *iso* harbored enterocytes of about 22μm thickness (the midgut epithelial thickness of wild-type flies feeding on food was roughly similar to that of flies feeding on sucrose solution when compared side-by-side). At 3 h p.i., when the cytoplasmic purge had occurred, the enterocytes had remarkably lost some 15μm thickness (median of 7μm) (Figure 2F). At 16 h p.i., while control flies had recovered their size (median of 25.5μm), enterocytes from NP > *acs* RNAi *iso* remained thin (median of 10.5μm), not different from *Sm*Db11 at 3h (lm, p = 0.1023).

The silenced *acs* full recovery phenotype 48h post-infection may reflect a hypomorphic effect of RNAi. We therefore generated a null *acs* mutant by CRISPR-Cas9, which also displayed a delayed recovery phenotype (Figures 2G, S1G, and S1H), indicating that the absence of *acs* can be compensated, albeit slowly.

All together, these results show that *acs* encodes an amino acid transporter induced in the gut upon infection and that it is required in enterocytes for a fast recovery of the gut epithelium after the cytoplasmic purge.

### A dynamic expression of ACS transcripts during infection

High-throughput expression data from FlyBase indicate that *acs* is expressed in the adult midgut, but it is also very highly expressed in the Malpighian tubules. In addition, data from Flygut-*seq* document an expression of *acs* in the gut visceral muscles.^18^ To visualize the tissues and the cell types in which *acs* is expressed, we used as a reporter a *UAS-GFP* transgene crossed to an *acs* CRISPR Knock-in mutant we have generated in which the entire CDS region of *CG1139/acs* has been deleted and replaced by Gal4 coding sequences (Figure S3A). Unexpectedly, although we observed the expression of *acs* in some enterocytes and mostly in the posterior midgut region, we also found it in cells with a shape characteristic of progenitor cells, in control or infection conditions (Figure S3B). Of note, the knock-in line may have deleted some regulatory elements present in the coding regions or introns and may therefore only partially reflect the endogenous expression of *acs*. Also, the transcript stability of Gal4 may differ from that of the endogenous *acs* gene.

**Figure 3:**
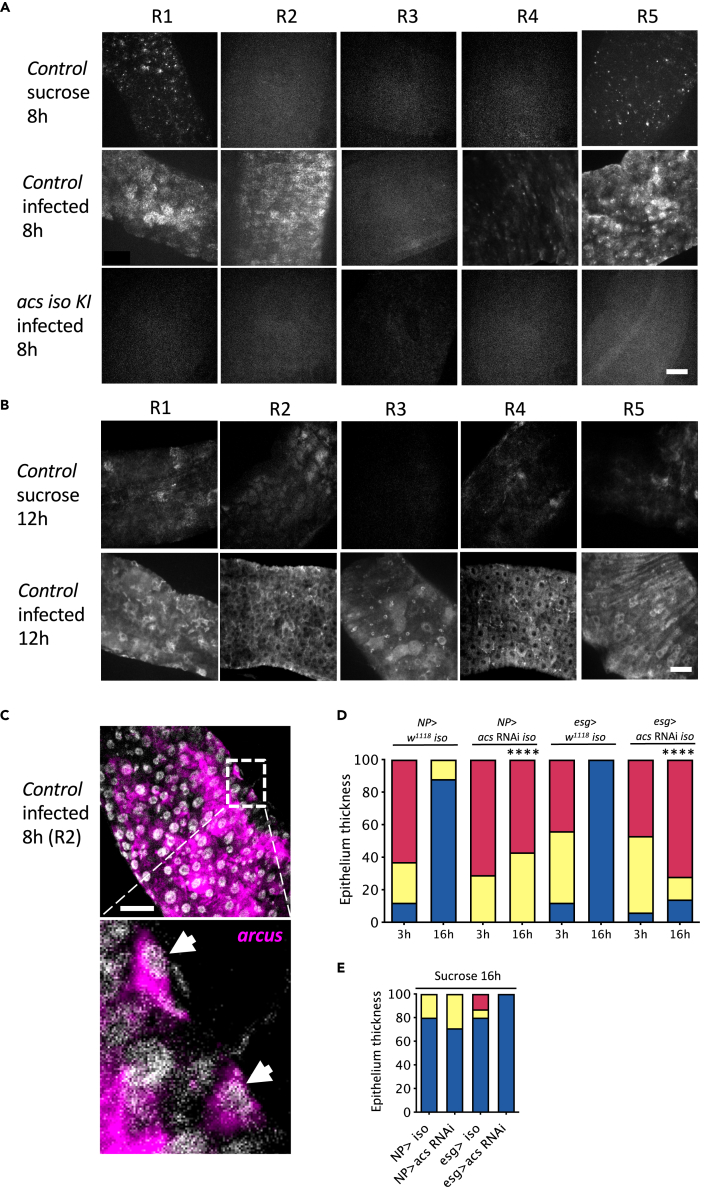
*acs* expression is dynamic after the ingestion of *Sm*Db11 bacteria (A and B) *acs* dynamic expression was revealed by *in situ* hybridization using the RNAscope technique on time-matched uninfected and *Sm*Db11 infected midguts at 8 and 12 h. The *acs* iso KI mutant is used as a negative control to probe the specificity of the signal. Scale bars 50μm. (C) *acs* is expressed in progenitor cells that are identified through their basal position and small size of the nuclei (arrowheads). Scale bar 50μm. (D and E) *acs* was knocked down in the enterocytes (*NP* > *acs* RNAi iso) or in the ISCs (*esg> acs* RNAi *iso*) and flies were infected with *Sm*Db11 (D) or exposed to sucrose (E). % epithelium thickness categories were assessed at 3h and 16h (chi-square, ∗∗∗∗ = p < 0.0001, comparisons at 16h between knocked down flies and corresponding controls crossed with *w*^*1118*^*iso*). Qualitative quantification of intestines according to their epithelial thickness: Thin-red, Semithin-yellow, Thick-blue (n = 24–30).

To verify the expression pattern of *acs* transcripts at different stages of the infection, we also performed *in situ* hybridization using the RNAscope technique. In control conditions (flies feeding on sucrose for 8 h), we observed some expression in scattered cells found mostly in the R1 and R5 regions, that is, the proximal and distal parts of the midgut. In contrast, the signal was stronger in these regions 8h p.i. and extended into the R2 region and to a lesser extent (weaker signal) in the R4 region. The hybridization was specific since no signal was detected in null *acs* KI mutants (Figure 3A). By 12h p.i., *acs* transcripts were observed throughout the midgut, including the R3 region (Figure 3B). Thus, the *acs* expression pattern is dynamic during infection and accounts for the increased steady-state expression measured initially by RT-qPCR (Figure 2B).

To genetically assess the relevance of the *acs* expression pattern, we knocked down its expression by RNAi in a tissue-specific manner. Whereas silencing *acs* ubiquitously (*ubi-Gal4-Gal80*^*ts*^) led to an impaired recovery of the intestinal epithelium thickness, this phenotype was not observed upon silencing its expression in the fat body (*yolk*-*Gal4*), in the Malpighian tubules (*uro*-*Gal4*), in the visceral muscles (*how*-*Gal4*), and in the enteroendocrine cells (*pros*-*Gal4*) (Figures S3C–S3E). A strongly impaired recovery phenotype was also observed upon silencing *acs* in progenitor cells (*esg*-*Gal4*) in keeping with *in situ* hybridization data (Figures 3C–3E). These results suggest that *acs* is also required in the progenitor cells of the gut epithelium during recovery. Alternatively, enterocytes newly differentiated during the five to seven days RNAi induction period silence the induced expression of the transporter due to retention of the interfering *acs* dsRNAs in those cells. Future studies will determine whether this dynamic pattern of *acs* transcripts is mediated through transcriptional control or progressively enhanced transcript stabilization, a second possibility more in keeping with the unexpected data obtained with the knock-in reporter transgene.

### ACS is required for increased fitness upon infection

We then assessed whether the impaired recovery phenotype might impact the fitness of the *acs-*silenced flies by monitoring them in several assays. Negative geotaxis assay did not reveal any difference between the silenced and control lines (Figure S4A). However, when chronically ingesting *SmDb11*, *acs*-silenced and mutant flies succumbed faster than control flies (Figures S4B and S4C). In addition, we found an increased bacterial burden in the crop and in the midgut of infected flies, even though the silenced flies did not ingest more food or defecate less after the infection (Figures S4D–S4G). Egg laying was also affected as the mutant flies did not present the increase in egg deposition observed in the control flies after infection (Figure S4H). Altogether, these results suggest that *acs* is required for increased fitness after infection, a likely indirect effect of its role in the timely recovery of the epithelium thickness.

### ACS localizes to the basal part of the gut enterocytes and is required for the retrograde transport of amino acids

**Figure 4:**
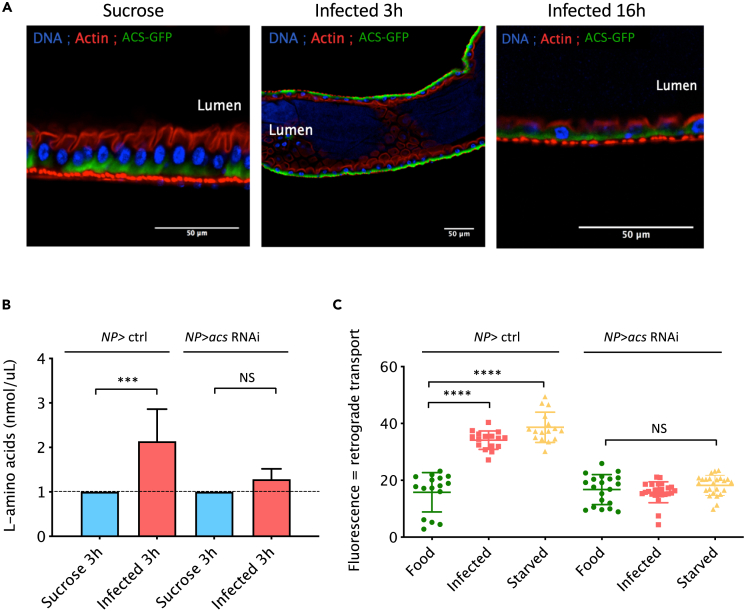
ACS localizes to the basal side of the enterocytes in the midgut epithelium and is required for the retrograde transport (A) Confocal pictures of dissected midguts from *NP>UAS-acs*-*GFP* flies exposed to sucrose or to *Sm*Db11 for 3h and 6h. Blue:DNA; Red:actin; Green:ACS-GFP; n = 30. (B) Quantification of free amino acids in the hemolymph of *NP >* ctrl and *NP > acs* RNAi 3h after sucrose exposure or *Sm*Db11 infection. Bars represent the mean and the error bars display Standard Deviation of the data (one-way ANOVA, ∗∗∗ = p < 0.001, n = 3 × 20 hemolymphs). (C) Flies were injected with 50 μM AHA and exposed to regular food, *Sm*Db11 (infected) or water (starved). Midguts dissected 6h later and fluorescence from the incorporated amino acid was assessed by staining with an alkyne probe and measured using ImageJ. The median bars represent the mean while the error bars display Standard Deviation of the data(*one-way ANOVA*, ∗∗∗∗ = p < 0.0001, n = 16–24). Stainings shown in Figure S2B.

ACS has previously been shown to be localized at the cell surface and on the late endosomal and lysosomal surfaces in *Drosophila* S2 cells, as well as in the larval fat body.^17^ When its reporter transgene was expressed in enterocytes, the functionally active ACS-GFP fusion localized to the basal labyrinth region of the cell that allow intensive exchanges of enterocytes with the hemolymph^19^ in uninfected and infected flies (Figure 4A).

Unexpectedly, the free amino acid peak measured in the hemolymph of control flies 3 h post-infection was not detected in *NP* > *acs* RNAi flies (Figure 4B). Strikingly, the AHA methionine analogue was not taken up by the gut epithelium of these silenced flies after infection or starvation thus directly showing that ACS is required for the retrograde transport of amino acids into the intestinal epithelium (Figures 4C and S2B). These results suggest that ACS may act both directly and indirectly to support the retrograde transport to the gut epithelium.

### ACS is not required cell-autonomously for the recovery from the cytoplasmic purge

**Figure 5:**
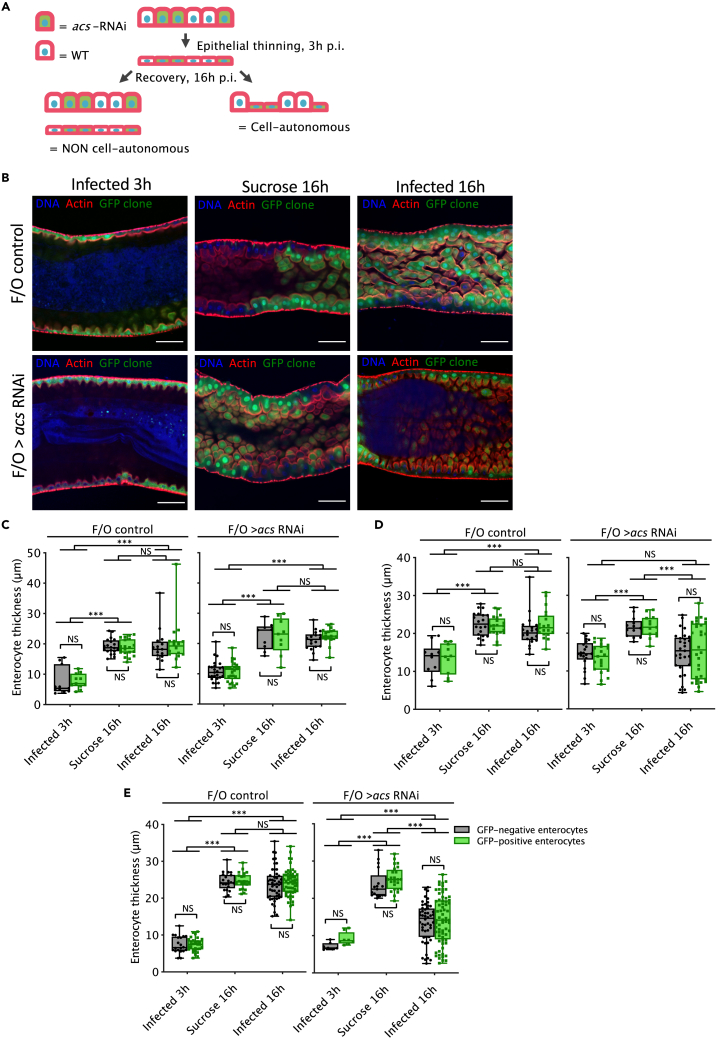
ACS is required non cell-autonomously for the recovery of the intestinal epithelium after *Sm*Db11 infection Clonal analyses performed using escargot Flp Out (*esg*F/O) system. (A) *w;esgGal4,tubGal80ts UAS-GFP*;*UAS-flp Act>CD2>Gal4* were crossed to *UAS-acs*-RNAi flies to generate clones of enterocytes expressing both GFP and RNAi against *acs* (F/O *acs*). A cell-autonomous mechanism implies that RNAi and wild type enterocytes recover at different rates, presenting a difference between their size 16h post-infection. (B) Confocal pictures of dissected midguts from F/O control or *acs* F/O flies at 3h and 16h post-infection and at 16h post sucrose exposure. Clones had been generated for 72h. Blue:DNA; Red:Actin; Green:*acs* RNAi enterocytes GFP positive. Scale bar = 50μm. Quantification of GFP-positive and negative clones for each condition is represented in (E). (C–E) Clones had been generated for 24h, 48h, and 72h, respectively; the middle bar of box plots represents the median and the upper and lower limits of the boxes indicate, respectively, the first and third quartile; the whiskers define the minima and maxima. (*lmer*, ∗∗∗ = p < 0.001, n = 4–7 midguts, merge of two independent replicates; NS: not significant).

Whereas ACS localizes to the basal part of enterocytes in keeping with a direct function in taking up some amino acids from the hemolymph, the absence of an increased release of amino acids in the hemolymph was however perplexing as it suggested a signaling function for ACS. We therefore performed a clonal analysis in the gut epithelium using the escargot Flip Out technique (*esg*F/O) that marks with GFP silenced cells of a clone initially generated in progenitor cells^20^ (Figure 5A). We generated clones for 24, 48 or 72 h resulting in a variable proportion of silenced epithelial cells of the midgut, ranging from a limited number of clones at 24h to a much more extended proportion at 72h. Importantly, the extent of recovery between wild-type cells and *acs-*silenced (GFP-expressing) cells was similar in the three conditions, suggesting a non cell–autonomous function for *acs* (Figures 5B–5E). However, the midgut recovery was similar to that of uninfected controls when clones had been induced for 24 h (Figure 5C). In contrast, the recovery was impaired when the clones had been generated for 48 or 72 h (Figures 5D and 5E) but not as strongly as when *acs* silencing was driven in all enterocytes (compare Figure 2F to Figures 5D and 5E). Thus, the extent of recovery roughly correlates inversely to the proportion of *acs-*silenced epithelial cells.

### The insulin signaling pathway and the transcription factor myc are required for the timely recovery of the gut enterocytes upon infection

**Figure 6:**
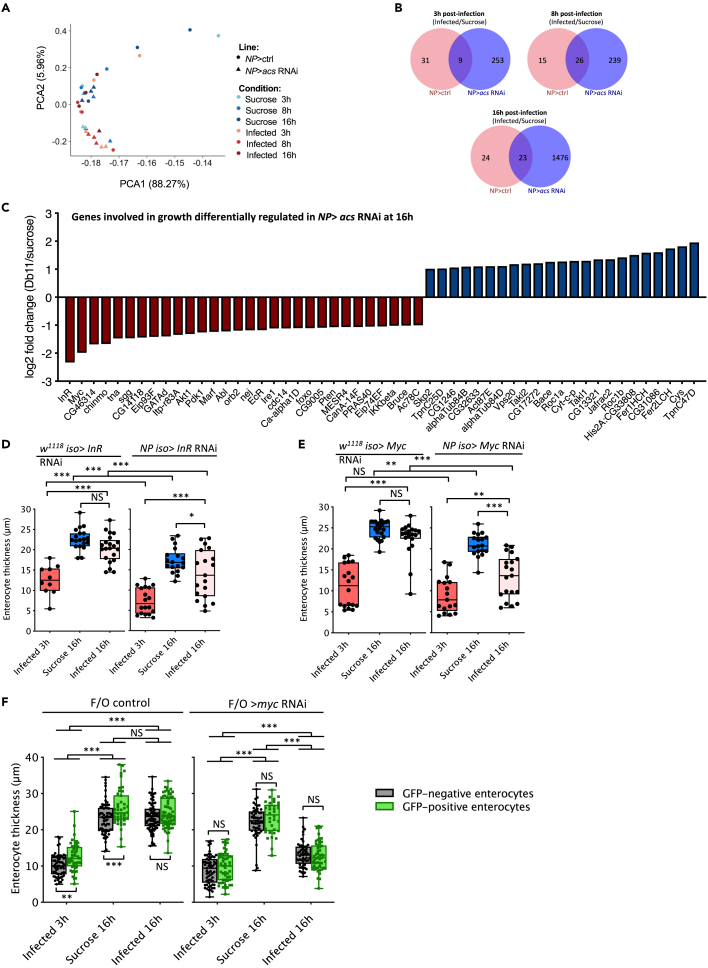
InR and the transcription factor *Myc* is required for the recovery of the gut enterocytes after infection *NP >* ctrl and *NP > acs* RNAi were exposed to sucrose or infected with *Sm*Db11 for 3h, 8h and 16h. RNA-seq was performed on dissected midguts. (A) Principal component analyses for gene expression of different samples. (B) Venn diagram for each time-point comparing genes differentially expressed (fold-change≥2) in *NP >* ctrl and *NP > acs* RNAi after infection, relative to sucrose. The scattering of the *NP* > ctrl data points (A) may limit the power of the analysis and hence lowering the number of significantly differentially regulated genes displayed in the pink circles. (C) Genes involved in growth that are up- or down-regulated in *acs* RNAi 16h after infection (relative to sucrose). All genes shown on the graph are differentially expressed with a log2-fold change≥2 and an adjusted p value≤0.05 (see also Table S6). (D and E) (D) *InR* and (E) *Myc* were knocked down in the gut enterocytes (*NPiso*> *InR* RNAi and *NPiso*> *Myc* RNAi) and compared with the respective controls for the recovery of the gut enterocytes thickness 16h upon infection or exposure to sucrose. (F) Midguts were dissected from F/O control or *Myc* F/O flies at 3h and 16h post-infection and at 16h post sucrose exposure. Thickness of GFP-positive and negative clones for each condition was quantified. Clones had been generated for 72h. D-F: the middle bar of box plots represents the median and the upper and lower limits of the boxes indicate, respectively, the first and third quartile; the whiskers define the minima and maxima (*lmer*, ∗∗∗ = p < 0.001, ∗∗ = p < 0.01, n = 10–22 midguts).

To understand how ACS functions, we performed an RNA-seq analysis in midguts of control flies or flies for which *acs* was silenced in the enterocytes at 3h, 8h, and 16h after *Sm*Db11 infection. We performed a principal component analysis (PCA) and observed a clear-cut difference on the PCA2 axis between noninfected and infected *NP > acs* RNAi flies, especially at 8h and 16h p.i. This difference was less pronounced between uninfected and infected wild-type (Figure 6A). Accordingly, more genes were differentially expressed in *NP > acs* RNAi than in wild-type, when comparing the infected samples to the corresponding sucrose controls (Figure 6B). KEGG pathway enrichment analysis did not reveal a differential enrichment of a particular category between control and *acs-*silenced flies, not even at 16h after infection for which we found many categories being enriched in the NP > *acs* RNAi flies (Figure S5A). However, the hierarchical clustering revealed that NP > *acs* RNAi flies 16h p.i. had many genes down- and up-regulated and did not cluster with the other samples (Figure S5B). Thus, this analysis revealed that ACS has an important impact on the physiology of the midgut that is likely, however, to be indirect since the midgut epithelial cells of 16 h wild-type flies have recovered their initial morphology.

As the analyses above failed to provide us with significant insights, we decided to employ another method to help us identify gene categories enriched upon infection between the wild-type and *NP > acs* RNAi. Therefore, we performed a Gene Set Enrichment Analysis (GSEA) for all the time-points comparing control and mutant knock-down flies to each other.^21^ With respect to the gene sets that were significantly up-regulated in the wild-type compared to the *acs* KD flies, we focused on the 8 and 16h time-points, the period during which the intestinal epithelium recovers its initial shape and thickness. Strikingly, at 8h we found an enrichment for genes known to be involved in the positive regulation of organ growth, positive regulation of translation, and genes required for lamellipodia assembly (Figure S5C). Genes from the latter category were also up-regulated in the wild-type at 16h, like the related set of genes involved in actin nucleation (Figure S5D). Whether these two gene sets play a role in the formation of the basal labyrinth of enterocytes remains to be determined.

Next, we extracted the expression levels of growth-related genes from the RNAseq dataset and compared infected-*acs* RNAi flies versus their corresponding sucrose control at 16h when wild-type enterocytes have recovered and *acs* enterocytes remain thin (Figures 6C and S5E (3h and 8h p.i.)). Several genes that are down-regulated in the infected *acs-*silenced midguts relative to uninfected controls are related to the Insulin pathway (*InR, Akt1, Pdk1, Dilp3, foxo, PTEN, PRAS40*), cellular growth (*Myc*), and *shaggy* (*sgg*), a link between insulin signaling and MYC expression. Interestingly, silencing either the *InR* or *Myc* genes led to a significantly impaired recovery of enterocyte thickness (Figures 6D and 6E). Of note, both genes are also involved in regulating the basal size of enterocytes (compare sucrose 16h in control vs. silenced flies). The already thin *InR*-silenced enterocytes under basal conditions may account for the apparently higher partial recovery observed for this line as compared to *Myc*-silenced enterocytes. Consistent with an absolute requirement of insulin signaling for enterocyte growth, we failed to obtain *InR* null-mutant enterocytes in clonal analysis. Counterintuitively, *Myc* steady-state transcripts levels were found to be decreased upon infection at both 8 and 16h pi. Whereas this difference was significant for *acs-*silenced flies at both 8 and 16h pi., it was no longer significant at 16h pi. for the control flies, leading thereby to a relatively increased expression of *Myc* in wild-type vs. *acs*-silenced flies at 16h p.i. (Figure S5F)*.* Thus, the levels of *Myc* may remain somewhat higher during recovery in wild-type as compared to the *acs* mutant enterocytes. In keeping with this finding, an *esg*-F/O clonal analysis with a *Myc* RNAi transgene revealed a non cell-autonomous phenotype similar to that of *acs*, which also led to an impaired recovery at 16h p.i. (Figure 6F).

The mTOR pathway is a major regulator of cell growth and ACS has been reported to interact with TOR in the fat body.^17^ Immunohistology stainings of midguts with antibodies against the phosphorylated form of 4EBP (P-4EBP), a direct TOR target, revealed an absence of signal in *acs-*silenced enterocytes 16h but not 3h pi. (Figure S6A). However, data obtained by directly silencing *TOR*, *S6K* or *Tsc2* yielded ambiguous results with a mild impaired recovery phenotype observed both for *TOR* and its negative regulator *TSC2* and a phenotype observed with silencing of *S6K* but not with a dominant negative allele (Figures S6B–S6E).

The EGFR pathway has been reported to promote enterocyte growth independently of Insulin and TOR signaling through the promotion of endoreplication in enteroblasts and newborn enterocytes after a *Pseudomonas entomophila* infection.^22^ Silencing *EGFR* expression in enterocytes did not affect the thickness of enterocytes under basal conditions but led to a mildly impaired recovery after exposure to *SmDb11* (Figure S6F). In contrast, targeting the E2F transcription factors that regulate endocycling did not affect recovery (Figures S6G and S6H). Finally, *Atg9* is also involved in regulating the thickness of enterocytes by negatively regulating the TOR pathway.^23^ As expected, the intestinal epithelium was thicker under basal conditions (sucrose) when *Atg9* was silenced in enterocytes. The silenced enterocytes underwent a thinning of similar extent than wild-type controls and regained their original size 16h p.i. (Figure S6I).

In conclusion, the Insulin signaling pathway and *Myc* are involved in the regrowth of purged enterocytes and ACS appears to promote the recovery of the steady-state expression of *Myc* mRNA that is strongly reduced early on during the infection.

## Discussion

The links between nutrition and intestinal epithelium homeostasis have been documented in the Drosophila model in the past decade.^22^^,^^23^^,^^24^^,^^25^^,^^26^ Here, we demonstrate that the recovery of enterocyte shape and volume that follows their extensive thinning resulting from the purge of their apical cytoplasm upon exposure to *SmDb11* pore-forming toxin does not involve the absorption of nutrients during or after the challenge. Rather, a reversion of metabolic fluxes from the organism to the intestinal epithelium is likely to occur, as documented by its uptake of an MET analogue injected in the hemocoel of the fly. We have identified the amino acid transporter Arcus/CG1139, known to transport ALA, PRO, and GLY, as being required for the retrograde transport of this MET analogue, and show that ACS is required for the timely regrowth of thinned enterocytes that may lose up to 90% of their volume. The study of ACS reveals that the intestinal epithelium is metabolically integrated, being highly sensitive to amino acid composition imbalance despite the likely exchange of amino acids between neighboring enterocytes. Besides its transporter function, ACS may play a role in interorgan communication by sharply increasing the levels of amino acids in the hemolymph 3 h p.i.

### A retrograde transport of metabolites

Whereas one of the major gut physiological function is the absorption of nutrients for subsequent export to the rest of the organism, enterocytes may actually import amino acids baso-laterally in interdigestive periods and permanently as regards mammalian crypt enterocytes.^27^^,^^28^ The metabolic toll exerted by the enterocyte purge as well as that occurring upon starvation in enterocytes is compensated by the retrograde transport of amino acids from the rest of the organism, and likely of other metabolites, at least in the case of the purge since major organelles and cytoplasm are extruded in the gut lumen. Upon starvation, the gut epithelium does not become as thin as upon *SmDb11* ingestion. Nevertheless, one may rationalize that the recovery of enterocyte functions is critical for survival so that the organism may quickly assimilate any food it might find while wandering. In the case of the purge, the simple delay of enterocyte recovery observed in *acs* mutants is likely related to the decreased fertility of females as well as the impaired survival upon infection, thereby testifying to the physiological relevance of fast recovery (Figures S4B, S4C, and S4H). As the purge corresponds to a defense reaction against ingested pathogens or xenobiotics, it is likely safer to reconstitute a normal epithelial barrier from internal stores rather than from potentially contaminated food.

ACS basal localization promotes the activity of this proton-assisted transporter: a low pH gradient from hemolymph to the basal side of the intestinal epithelium, peaking at 4.34 near the basal labyrinth, likely provides the proton-motive force for the import of amino acids.^29^ Thus, in contrast to other cell types, ACS may not necessarily function in the late endosomal/lysosomal compartment^17^ but rather at the membrane.

### ACS mediates enterocyte regrowth through the insulin signaling pathway and Myc

The fast recovery of the intestinal epithelium after thinning can only be ascribed to regrowth since ISC compensatory proliferation is not involved.^14^ An alternative would be an increased ploidy of enterocytes to facilitate faster growth.^22^^,^^25^^,^^26^ Whereas we did find a mild requirement for EGFR function in the recovery phase, its role in growth may not be related to its role in the control of the ploidy of enterocytes since silencing the genes encoding the E2F transcription factors required for endocycling did not reveal any impaired recovery phenotype. Rather, it appears that the Insulin Signaling pathway and possibly the mTOR pathway mediate the regrowth of enterocytes through MYC, a major regulator of growth.^30^^,^^31^^,^^32^^,^^33^^,^^34^^,^^35^^,^^36^ Several studies have established a link between CG1139/ACS and mTOR activation,^16^^,^^17^^,^^37^^,^^38^ yet our genetic data do not allow us to draw an unambiguous conclusion on the existence of this link in recovering enterocytes.

Our results are consistent with a model in which MYC activity is decreased during the purge and subsequently progressively returns to original levels of expression in an *acs*-dependent manner. Indeed, we note that the expression of *sgg* is up-regulated at 3h (thin epithelium) in *acs*-silenced flies (Figure S5E). As *sgg* encodes GSK3β that negatively regulates MYC stability,^39^^,^^40^ we infer that MYC levels will take longer to be reinstated in *acs* mutant flies. Likewise, *sestrin* (*sesn*) encodes a negative feedback mTOR pathway inhibitor that works through an AMPK/Tsc2 axis.^41^ Its expression is also significantly upregulated at 3 and 8h but not 16h in *acs* mutants (Figure S5E) suggesting that it leads to a sustained repression of the mTOR pathway when the intestinal epithelium is thin. The delayed intestinal epithelium recovery phenotype in *acs* mutants may result from an impaired capacity to reactivate the Insulin signaling and possibly the mTOR pathways in a timely manner.

Our study highlights the importance of *Myc* in the recovery process given the similarity of its phenotype to that of *acs*. Its expression is regulated both at the transcriptional and post-transcriptional levels.^42^ We note that cMyc protein stability can be indirectly regulated by amino acids levels via system L transport of large, branched, aromatic or neutral amino acids in murine T lymphocytes and natural killer cells, specifically through the SLC7A5 transporter.^40^^,^^43^ Whereas the *Drosophila SLC7A5* gene tested negative in our RNAi mini-screen, the *SLC7A7* (*minidiscs*) and SLC7A9 (*sobremesa*) transporter genes yielded a phenotype nearly as pronounced as that of *acs*. Thus, an in-depth study of these two transporters is warranted and it will be especially interesting to determine whether their phenotypes are mediated by MYC.

### ACS and homeostatic mechanism that counteract amino acid imbalance

ACS appears to function at several levels. Since it is required for the timely recovery of enterocyte shape and volume, it must be indirectly involved in the reverse metabolic imports not only of other amino acids but also of other basal metabolites to replenish the lost cytoplasm and organelles, a process that is likely coordinated. Indeed, ACS has been shown to transport ALA, GLY, and PRO. Thus, other amino acids, including methionine, are bound to be imported by other transporters. Candidates have been identified in our mini-screen (Figures S1B and S1C), with a prevalence of SLC7 family members transporting cations or belonging to the L-system as noted above. *slif* and *mnd* have been shown to be required for growth of the organism and imaginal discs respectively.^44^^,^^45^ MND also imports LEU into insulin-producing cells and regulates Dilp2 release and thus like SLIF plays a role in the systemic regulation of growth.^46^ It is also likely that other transporters fill in for ACS when mutated, albeit more slowly. Thus, when *acs* activity is missing, the likely decreased availability of ALA, GLY, and PRO likely limits the uptake of other metabolites in a regulatory process that remains to be delineated.

A second level of amino acid homeostasis throughout the midgut epithelium has been revealed by our clonal analysis in the midgut epithelium in which wild-type enterocytes fail to recover from the cytoplasmic purge to the same extent as *acs* enterocytes. Importantly, the level of recovery appeared to be approximately inversely proportional to the proportion of *acs* mutant cells in the epithelium. This correlation is unlikely to result from a persistence of the Arcus protein when clones are generated for a short time since the *acs* gene is initially expressed in only very few cells prior to infection. Whether clones have been generated for one or three days, the silencing of *acs* gene expression will occur upon the same time frame of the infection; only the number of epithelial cells in which *acs* will be silenced as its expression is induced will differ. This clonal analysis thus suggests that wild-type enterocytes are able to compensate the deficient uptake of ALA, GLY, or PRO in neighboring cells. The simplest hypothesis is to propose that amino acid can directly circulate between epithelial cells through gap junctions or alternatively by secretion and re-uptake by other amino acid transporters. A previous study in the posterior larval midgut did not reveal any electrical coupling between neighboring enterocytes, thus making unlikely the existence of gap junctions within this epithelium.^47^ However, the components of *Drosophila* gap junctions, the innexins, are expressed in the diverse types of the epithelial adult midgut^48^ and it will be interesting to determine whether the two major innexins expressed in enterocytes, Inx2 and Inx7, mediate the *acs* non cell-autonomous phenotype.

### A function for ACS in interorgan communication?

*acs* function is required for the surge of amino acid concentration in the hemolymph shortly after exposure to *SmDb11* (Figure 3C), before the strong induction of *acs* expression that occurs later on. The source of these amino acids has not been identified so far and hemolymphatic proteins are potential contributors that we failed to pinpoint reproducibly. As the increased concentration of amino acids in the hemolymph is temporally the first *acs*-dependent process we have identified, it may constitute the stimulus that coordinates the uptake of metabolites required for the regrowth of enterocytes.

Our transcriptomics analysis in wild-type guts did not allow us to identify obvious candidate signaling molecules. In *acs* mutants, hundreds of genes are differentially expressed upon *SmDb11* ingestion, which suggests that the resilience response is perturbed and must be adapted to rise to the challenge. Given the biochemical nature of ACS, its potential signaling functions are likely mediated indirectly.

The finding of a role for *acs* in the generation of the increased amino acid concentration is perplexing as in unchallenged control midguts *acs* appears to be expressed in very few cells that might act as sentinels. Whether these cells are also required for the expanding expression of *acs* throughout the midgut epithelium remains to be determined. We have been unable to determine whether *acs* is required for its own increased expression since the frameshift KO mutant did not produce transcripts detectable by RT-qPCR or *in situ* hybridization, likely because of nonsense-mediated mRNA decay. How *acs* expression is regulated will be a major issue to address in future experiments. It will also be important to question the specificity of this ACS function, *i.e.*, whether this phenotype is shared when mutating other transporters that appear to be also needed for recovery.

The size of a given cell type in an organ is usually rather uniform, implying there are inherent processes that regulate the control of final cell size that remain poorly understood at present.^49^ There are multiple mechanisms that contribute to the establishment of final cell size including nutrition, biogenesis, and most importantly cell division. Cytoplasmic extrusion as documented here provides a unique setting to study the regulation of cell size independently of most of the parameters cited above. Interestingly, we have recently established that the thickness of regrown enterocytes is slightly larger in enterocytes that have been exposed to *Sm*Db11 hemolysin. This study establishes that cell size does not appear to be regulated intrinsically at the enterocyte level but may be a property set up at the levels of the organ or of the whole organism.

### Limitations of the study

The present study has been performed under our conditions (food, exact temperatures, microbiota lacking *Acetobacteraceae*) and a limited number of genetic backgrounds. We have used our regular food for our experiments as a yeast-rich food we also use in our laboratory inhibits the apical cytoplasmic purge due to the presence of ethanol used to dissolve the methyl paraben preservative.^50^^,^^51^ The mini-genetic screen that identified *Arcus* relied on RNAi transgenic lines; except for *acs*, the other hits have not been validated using independent RNAi lines or mutants. The *acs* KI mutant deletes the whole locus, including the endogenous *acs* 3′ untranslated region, which may therefore affect the stability of the reporter transcripts as compared to the endogenous ones.

## STAR★Methods

### Key resources table

**Table:** 

REAGENT or RESOURCE	SOURCE	IDENTIFIER
**Antibodies**		

Fluorescein Isothiocyanate (FITC)	Sigma-Aldrich	#P5282
Texas-Red labeled phalloidin	Invitrogen TM	#T7471
Mouse anti-GFP antibody	Roche	#11814460001; RRID:AB_390913
Goat anti-mouse FITC antibody	Abcam	#6785; RRID:AB_955241
Rabbit anti-phospho-4EBP1 antibody	Cell Signaling	#2855; RRID:AB_560835
Goat anti-rabbit FITC antibody	Abcam	#6717; RRID:AB_955238
Vectashield	Vector Laboratories	H-1000
Vectashield with DAPI	Vector Laboratories	H-1200
Alexa Fluor 488 alkyne dye	Thermofisher	#A10267

**Bacterial and virus strains**		

*Serratia marcescens* Db11 *(Sm*Db11*)*	Flyg et al.^52^	N/A
*Serratia marcescens* 21C4 *(Sm*21C4*)*	Kurz et al.^11^	N/A

**Biological samples**		

Intestines	This study	N/A

**Chemicals, peptides, and recombinant proteins**		

Essential amino acids	Thermo Fischer Scientific	MEM Amino Acids Solution 50X #11130051
Non-essential amino acids	Thermo Fischer Scientific	MEM Non-Essential Amino Acids Solution 100X, #11140076
Chloramphenicol	Euromedex	3886-B
Streptomycin	Sigma-Aldrich	S6501
TRI Reagent RT	Molecular Research Center	TR118
Bromoanisole	Molecular Research Center	BAN, 98%,

**Critical commercial assays**		

iScript cDNA synthesis kit	Bio-Rad	#1708890
iQ TM SYBR Green Supermix kit	Bio-Rad	#1525121
L-amino acid quantitation kit	Sigma-Aldrich	#MAK002
Click-it AHA (L-azidohomoalanine) kit	Thermofisher	#C10102
Click-it Cell Reaction Buffer kit	Thermofisher	#C10269
FLIC system (FLIC-MCU+DFM)	Sable Systems Europe	N/A

**Deposited data**		

RNA sequencing WT 3h post-infection	Lee et al.^14^	GEO Bioproject: GSE60504
RNA sequencing WT 6h and 9h post-infection	This study	GEO Bioproject: PRJNA258425
RNA sequencing WT vs. acs-RNAi at 3h, 8h and 16h post-infection	This study	GEO Bioproject: PRJNA821079

**Experimental models: Organisms/strains**		

*Drosophila melanogaster wA5001*	Thibault et al.^53^	N/A
*D. melanogaster w*^*1118*^*iso*	Laboratory of Luis Teixeira (Ryder et al.)^54^	N/A
*D. melanogaster NP-Gal4-tubGal80*^*ts*^*(*also known as Myo31D)	Cronin et al., 2009; Nehme et al., 2007^12^^,^^13^	N/A
*D. melanogaster NP-Gal4-tubGal80*^*ts*^*iso*	This laboratory	N/A
*D. melanogaster Ubi-Gal4tubGal80*^*ts*^	This laboratory	N/A
*D. melanogaster Yolk-Gal4*	This laboratory	N/A
*D. melanogaster prospero-Gal4*	Bloomington stock center	#84276
*D. melanogaster esg-Gal4Gal80*^*ts*^	Micchelli&Perrimon^55^	N/A
*D. melanogaster how>Gal4*	Bloomington stock center	#1767
*D. melanogaster mex1>Gal4*	Bloomington stock center	#91368
*D. melanogaster uro-Gal4*	Laboratory of Bruno Lemaitre (Li et al.)^56^	N/A
*D. melanogaster* w; *esgGal4tubGal80*^*ts*^*UAS-GFP; UAS-flp Act>CD2>Gal4*	Jiang et al.^20^	N/A
*D. melanogaster acs iso* CRISPR KO	Sino-French Hoffmann Institute and this laboratory	N/A
*D. melanogaster acs iso* CRISPR KI	Well Genetics and this laboratory	N/A
*D. melanogaster arcus*-RNAi (*CG1139* RNAi)	VDRC	#102363
*D. melanogaster arcus*-RNAi iso	This laboratory	N/A
*D. melanogaster UAS-CG1139-GFP*	Gift from Prof. Deborah Goberdhan^17^	N/A
*D. melanogaster InR* RNAi	Bloomington Drosophila Stock Center	#51518
*D. melanogaster Myc* RNAi	Bloomington Drosophila Stock Center	#51454
*D. melanogaster S6K* RNAi	Bloomington Drosophila Stock Center	#42572
*D. melanogaster S6K-KQ*	Bloomington Drosophila Stock Center	#6911
*D. melanogaster TOR* RNAi	Bloomington Drosophila Stock Center	#33627
*D. melanogaster Tsc2* RNAi	Bloomington Drosophila Stock Center	#31776
*D. melanogaster Atg9* RNAi	Bloomington Drosophila Stock Center	#28055
*D. melanogaster E2f1* RNAi	VDRC	#108837
*D. melanogaster E2f2* RNAi	VDRC	#100990
*D. melanogaster Egfr* RNAi	VDRC	#107130

**Oligonucleotides**		

rp49 fw 5′GACGCTTCAAGGGACAGTATCTG-3′	This laboratory	N/A
rp49 rv 5′- AAACGCGGTTCTGCATGAG3′	This laboratory	N/A
*acs* fw 5′ACGTCAGCTTTTCGCAGGCCA-3′	This publication	N/A
*acs* rv 5′-ACAACGCAGCCAGGGTGGAC-3′	This publication	N/A
*myc* fw 5′ CAGTTCCAGTTCGCAGTCAA-3′	This publication	N/A
*myc* rv 5′ AGATAAACGCTGCTGGAGGA-3′	This publication	N/A

**Software and algorithms**		

ImageJ/Fiji software	Fiji software	https://imagej.nih.gov/ij/
GraphPad Prism v8.2.1	GraphPad software	https://www.graphpad.com/
CFX384 system	Bio-Rad	1855484
HISAT2	Kim et al.^57^	https://github.com/DaehwanKimLab/hisat2
Bowtie2	Langmead and Salzberg^58^	https://bowtie-bio.sourceforge.net/bowtie2/index.shtml
RSEM	Li et al.^59^	https://github.com/deweylab/RSEM
DEseq2 algorithm	Love et al.^60^	https://bioinformaticshome.com/tools/rna-seq/descriptions/DESeq2.html#gsc.tab=0
GSEA v4.1.0	Subramanian et al.^21^	https://www.gsea-msigdb.org/gsea/index.jsp

**Other**		

absorbent pads	Millipore	# AP1003700
DNBseq platform	BGI	N/A

### Resource availability

#### Lead contact

Further information and requests for resources and reagents should be directed to and will be fulfilled by the lead contact, Dominique Ferrandon (D.Ferrandon@ibmc-cnrs.unistra.fr).

#### Materials availability

• *Drosophila melanogaster* lines generated in this study are available upon request.
• This study did not generate any unique new reagents.

### Experimental model and study participant details

#### Drosophila melanogaster *husbandry*

*Drosophila melanogaster* flies were raised at 25°C and 60% humidity, 14h of daylight and fed a standard semi-solid cornmeal medium (6.4% (w/v) cornmeal (Moulin des Moines, France), 4.8% (w/v) granulated sugar (Erstein, France), 1.2% (w/v) yeast brewer’s dry powder (VWR, Belgium), 0.5% (w/v) agar (Sobigel, France), 0.004% (w/v) 4-hydroxybenzoate sodium salt (Merck, Germany)).

*acs* RNAi isso and NP iso were lines line isogenized to *w*^*1118*^ *iso* genetic background, as described in.^61^ The drivers were used either alone, crossed with *w*^*1118*^ *iso* as controls, or crossed with a RNAi or a reporter line. Crosses for Figures 5D, 5E, and S5B–S5I were performed by crossing the *NP-Gal4-tubGal80*^*ts*^ driver isogenized to *w*^*1118*^ *iso* (referred as NP *iso*) or *w*^*1118*^ *iso* to the RNAi line of interest. Crosses were performed at 25°C except for *Inr*, *Myc*, *Egfr*, *E2f1, E2f2* and *Atg9,* which were performed at 18°C. The F1 progeny of these crosses was then placed at 29°C with 70% humidity for 5 to 6 days in order to induce the expression of the *Gal4tubGal80*^*ts*^ transgenes. For clonal analysis, the Flp-Out line w; *esgGal4tubGal80*^*ts*^ *UAS-GFP; UAS-flp Act>CD2>Gal4* from Jiang et al.^20^ was used. *acs* knock-out mutant was generated by the Sino-French Hoffmann Institute CRISPR-Cas9 platform and comprises a 5bp deletion in the sequence causing a presumably truncated non-functional protein (Figures S1E and S1F). *acs* knock-in mutant was generated by deleting and replacing the entire CDS region of *CG1139/acs* by cassette of Gal4 in Well Genetics (Figure S2A). Both mutants were isogenized to *w*^*1118*^ *iso*.

3-6 days old females were used for the experiments, unless otherwise specified in the figure legend.

#### Serratia marcescens

*Serratia marcescens* strain Db11 (*Sm*Db11) was cultured on Luria-Bertani Broth (LB) agar plates with 100 μg/mL of streptomycin. The strain 21C4 *Sm*21C4^11^ was cultured on LB-agar plates with 20 μg/mL chloramphenicol. The solid plates were placed overnight at 37°C to obtain colonies. For liquid cultures, one bacterial colony was taken off the solid plate and inoculated into 200 mL of liquid LB. These cultures were kept overnight at 37°C with agitation.

### Method details

#### Infections

All infections were performed using a final bacterial OD 600 (Optical Density at 600 nm) of 10, except for the survival where OD = 1 was used. The OD was measured with a spectrophotometer and 50 mL of this solution was centrifuged at 4000 g/rcf for 10 minutes. The pellet was re-suspended in the appropriate volume of a 50mM sucrose solution containing 10% of LB, in order to reach a final OD = 10. 2 mL of this infection solution were added to two absorbent pads (Millipore AP1003700) that were placed at the bottom of medium-size vials (3.5 cm diameter). Twenty female flies of five to seven-day old were fed this infection solution, or sucrose 50mM as a control at 29°C.

For Figure 1A, essential amino acids (MEM Amino Acids Solution 50X, Thermo Fischer Scientific #11130051) were added to the infection solution to a final 1x concentration. The 1x solution is composed of: L-arginine hydrochloride (126.4 mg/L), L-cysteine (24 mg/L), L-histidine hydrochloride (42 mg/L), L-isoleucine (52.4 mg/L), L-leucine (52.4 mg/L), L-lysine hydrochloride (72.5 mg/L), L-methionine (15.1 mg/L), L-phenylalanine (33 mg/L), L-threonine (47.6 mg/L), L-tryptophan (10.2 mg/L), L-tyrosine (36 mg/L) and L-valine (46.8 mg/L). Non-essential amino acids (MEM Non-Essential Amino Acids Solution 100X, Thermo Fischer Scientific #11140076) were added to a final 1x concentration. The 1x solution is composed of: glycine (15 mg/L), L-alanine (17.8 mg/L), L-asparagine (26.4 mg/L), L-aspartic acid (26.6 mg/L), L-glutamic acid (29.4 mg/L), L-proline (23 mg/L) and L-serine (21 mg/L).

#### Fluorescent histochemical staining

Midguts were dissected in PBS and fixed for 30 minutes with 4% paraformaldehyde. Samples were washed three times with PBS-Triton X-100 0.1% (PBT 0.1%).

#### Actin staining

For actin staining midguts were incubated for 1h30 at room-temperature or overnight at 4°C in 10 μM Fluorescein Isothiocyanate (FITC) (Sigma-Aldrich #P5282) or Texas-Red labeled phalloidin (Invitrogen TM #T7471). Samples were then washed three times with PBT 0.1%.

#### GFP staining

To visualize the CG1139/ACS-GFP fusion protein, midguts were incubated with 1:500 anti-GFP (mouse) antibody (Roche #11814460001) for 3h. Samples were then washed three times with PBT 0.1% and incubated with a secondary goat anti-mouse FITC antibody (Abcam #6785) for 1h30. In the case of a co-staining with phalloidin and antibodies, the primary antibody was added alone for 3h, followed by 2h of incubation with a mix containing phalloidin and the secondary antibody. The visualization of *acs* KI> *UAS-GFP* was directly done without antibody staining.

#### Phospho 4EBP staining

Midguts were blocked 2h in PBT 0.1% with 2% bovine serum albumin (BSA). P-4EBP was detected with the anti-phospho-4EBP1 rabbit antibody from Cell Signaling (#2855). Midguts were incubated for 3h with 1:200 of the P-4EBP antibody, washed three times with PBT 0.1% and incubated with a secondary goat anti-rabbit FITC antibody (Abcam #6717).

All samples were mounted on diagnostic microscope slides (Thermo Fisher Scientific) with Vectashield plus DAPI (Vector Laboratories). Samples were observed using a LSM780 confocal microscope (Zeiss) or in Axioskop 2 microscope (Zeiss). All images were analyzed with the ImageJ/Fiji software.

#### RTqPCR

RNA was extracted from 5 or 10 midguts (without crop and Malpighian tubules) in triplicates. Midguts were crushed into 100 μL of TRI Reagent RT (Molecular Research Center) with 5% of bromoanisole (BAN, 98%, Molecular Research Center). Samples were vortexed, incubated 5 minutes at room temperature and centrifuged 10 minutes, 15 000rcf/g at 4°C. The upper phase of the samples was collected, mixed with 350 μL of isopropanol and vortexed. Tubes were centrifuged at 18 000rcf/g for 15 minutes at 4°C. The pellet was washed twice with 500μL of ethanol 70% and dried. RNAs were then re-suspended in 15μL of MilliQ water (Millipore). 1 μg of RNA was then used to generate cDNA by reverse transcription, using the iScript cDNA synthesis kit (Bio-Rad #1708890). The quantitative Polymerase Chain Reaction (qPCR) was performed with the Bio-Rad iQ TM SYBR Green Supermix kit and data were analyzed with the CFX384 system (Bio-Rad). mRNA quantitation was done by normalizing the amount of RNA detected for the gene of interest, with the control rp49 mRNA levels. The relative gene expression was calculated by normalizing the values obtained from sucrose fed flies (controls) versus challenged ones (infected with *Sm*Db11). Primers used for rp49 were the forward 5′GACGCTTCAAGGGACAGTATCTG-3′ and the reverse 5′- AAACGCGGTTCTGCATGAG3’. Primers used for *acs* were the forward 5′ACGTCAGCTTTTCGCAGGCCA-3′ and the reverse 5′-ACAACGCAGCCAGGGTGGAC-3’. Primers used for *myc* were the forward 5′ CAGTTCCAGTTCGCAGTCAA-3′ and the reverse 5′ AGATAAACGCTGCTGGAGGA-3’.

#### Quantification of free amino acids

Free amino acids were measured using the L-amino acid quantitation kit from Sigma-Aldrich (#MAK002). Females flies were either starved on sterile water, kept on their normal food, fed sucrose 50mM or fed the usual infection solution for 3, 6 and 12h. The hemolymph from 20 flies were collected into 10μL of the kit buffer and processed according to the manufacturer’s instructions. Samples were run in biological duplicates.

#### Click-it assay

The Click-it reaction is a non-radioactive technique that allows the detection of nascent proteins. AHA corresponds to a modified amino acid mimicking methionine that is normally incorporated into proteins. The detection is based on the reaction between an azide group on the modified amino acid and the alkyne group of a fluorophore. The Click-it assay was performed with the Click-it AHA (L-azidohomoalanine) kit from Thermofisher (#C10102), the Alexa Fluor 488 alkyne dye (#A10267) and the Click-it Cell Reaction Buffer kit (#C10269). The reagents were prepared according to the manufacturer’s instructions. 50μM of AHA diluted in PBS was injected in the hemolymph of female flies, in order to observe we could later detect its presence in the midgut of flies. Flies were placed on different conditions: i) their normal food ii) sterile water iii) sucrose 50mM iv) *Sm*Db11 diluted in sucrose 50mM. Midguts were dissected in PBS 6h post-treatment, fixed for 20 minutes in 4% paraformaldehyde and washed with PBT 0.1%. To detect the injected AHA, midguts were stained for 30 minutes (protected from light) with 0.5 mL of the following mix: 437.5μL of 1X Click-it Reaction Buffer, 10μL of CuSO 4, 50μL of Click-it Buffer Additive and 2.5μL of 1mM Alexa Fluor 488 alkyne. Midguts were washed three times with PBT 0.1% for 15 minutes and mounted on microscopy slides as previously described. Samples were immediately observed at the confocal microscope and the fluorescence intensity was measured with the ImageJ software.

#### Fitness parameters

The negative geotaxis assay was performed as described in Linderman et al.^62^: ten flies per vial were either infected with *Sm*Db11 OD600 = 10 or fed in 50mM sucrose as a control. After 3h or 16h of feeding, they were transferred in the vials designed for the assay without anesthesia. A line was drawn at 8cm from the bottom of the tube. The number of flies crossing this line after 5 and 10 seconds was measured and plotted on a graph.

Survival assays were performed using 10 to 20 flies per vial in triplicates. The experiments were conducted with five to seven-day old adult females at 29°C with 70% humidity. The flies were fed either sucrose 50mM as a control or infected with an OD600 = 1 of *Sm*Db11. Two absorbent pads were placed at the bottom of medium-size vials and 2mL of sucrose or bacterial solution were added to the filters. Each day, flies that were alive were counted and 200μL of 100mM sucrose was added to the vials.

Bacterial loads in the midguts or crop were accessed 16h post *Sm*Db11 infection (OD600 = 10). Guts were dissected and pools of 3 midguts and crops were homogenized in 100μl of PBS1x and serial dilutions were performed to plate. Extracts were plated in LB agar containing 100μg/mL of streptomycin and incubated at 37°C overnight to count total number of colonies.

Food intake was accessed either by quantifying the level of ingested blue dye^63^ or using the FLIC system.^64^ To access the level of ingested blue dye, flies were either fed in 50mM sucrose or *Sm*Db11, in a solution containing 5% of standard food blue dye. After 16h females were homogenized in 50μl of PBS1x and samples were centrifuged at maximum speed for 10min. Absorbance was accessed using the Varioscan and the wavelength was measured at 625nm. The absorbance for each sample was calculated to the relative controls, where flies were subjected to the same conditions but without blue dye. Each sample corresponds to a pool of 10 females. To access the food intake using the FLIC system we placed one fly per well feeding either in sucrose or *Sm*Db11 solution (OD600 = 10). 12 females were accessed per condition in each experimental replicate. Number of events (groups of contiguous licks) was considered as a read-out for the food intake.

Defecation assay was adapted from a previously described assay,^65^ by feeding 10 females per vial for 16h either in 50mM sucrose or in *Sm*Db11 (OD600 = 10) in a solution containing 5% of standard food blue dye. Fecal spots on the vial walls were counted.

Egg laying was measured using 1–2 days old *w*^*1118*^ *iso* and *acs* knock-out mutant females. Females and males were exposed for 16h to either 50mM sucrose or to *Sm*Db11 (OD600 = 10), and after single females together with 2 males were transferred to vials with standard fly food for 24h, and flipped to new vials for another 24h. Number of eggs ovoposited was counted at 24h and at 48h after infection.

#### RNA sequencing

To analyze genes differentially regulated in NP > ctrl and NP > *acs* RNAi flies after infections, flies were infected with *Sm*Db11 or exposed to sucrose and midguts were dissected 3h, 8h, or 12h post infection. Each sample comprised 10 midguts (without crop and Malpighian tubules) and three replicate samples were generated per condition.

Samples were sent to BGI and RNA was sequenced using the DNBseq platform. We used the Agilent 2100 Bio analyzer (Agilent RNA 6000 Nano Kit) to perform the total RNA sample quality control RNA concentration, 28S/18S, and the fragment length distribution. Firstly, we removed the reads mapped to rRNAs and obtained raw data; then, we filtered out the low quality reads (More than 20% of the bases qualities are lower than 10), reads with adaptors and reads with unknown bases (N bases more than 5%) to get the clean reads. We assembled those clean reads into Unigenes, followed with Unigene functional annotation and calculated the Unigene expression levels and SNPs of each sample. Finally, we identify DEGs (differential expressed genes) between samples and performed clustering analysis and functional annotations. After filtering the reads, clean reads were mapped to reference genome using HISAT2^57^ and the average mapping ratio with reference genome was 94.08%. Clean reads were mapped to reference transcripts using Bowtie2,^58^ and gene expression level was calculated for each sample with RSEM.^59^ Based on the gene expression level, we used DEseq2 algorithms to identify the DEG (Differentially expression genes) between samples or groups. With DEGs we performed Gene Ontology (GO) classification, KEGG pathway classification and functional enrichment. We calculated false discovery rate (FDR) for each p value, in general, the terms which FDR not larger than 0.01 are defined as significant enriched. We extracted here genes involved in cell growth where we identified genes involved in insulin signaling and the transcription factor myc.

The average mapping ratio with reference genome was 94.08%, the average mapping ratio with gene was 80.06% and a total of 17,003 genes were detected.

Gene Set Enrichment Analysis (GSEA) was performed as described using GSEA v4.1.0.^21^ We used a gene set library from the FlyEnrichr that distributes the genes according to biological processes from their Gene Ontology, “GO_Biological_Process_AutoRIF_Predicted_zscore”.^66^^,^^67^

### Quantification and statistical analysis

#### Classification of gut epithelium thickness

To determine the level of thinning and the recovery capacity, we used either qualitative or quantitative analysis. For qualitative analysis, midguts were classified into three different categories according to their epithelial thickness. Thick epithelium, which we represent in blue, corresponds to cells with the normal size, (around 20μm in length), with a clear dome shape characteristic of the intestine. Thin epithelium, which we represent in red, corresponds to cells that are very thin (around 5μm) and the normal dome shape of the cells is not observed. Semi-thin epithelium, which we represent in yellow, corresponds to cells with intermediate thickness (around 13μm), where the dome shape is still not completely defined. For quantitative analysis, pictures of midguts were acquired using Fluorescence Axioskope Zeiss and the length of gut enterocytes was measured using ImageJ/Fiji software. The measure for each midgut corresponds to the average of the measure between 10 enterocytes. Measures were taken every 5 to 10 enterocytes.

#### Statistical analyses

Statistical analyses were performed using GraphPad software Prism 6 and R. Statistical tests used are stated in the correspondent figure legends. For qualitative analysis of epithelium thickness, we used chi-square statistical tests. Survivals were analyzed using mixed effects cox models^68^; available at https://CRAN.R-project.org/package=coxme. Experiments including different factors were analyzed using Linear models (lm) or linear mixed-effect models (lmer, package lme4)^69^ in order to include the different factors of the experiment, such as the fly line or the treatment, and to include random factors such as the experimental replicates. Significance of interactions between factors was tested by comparing models fitting the data with and without the interactions, using analysis of variance (anova). Models were simplified when interactions were not significant. Pairwise comparisons of the estimates from fitted models were analyzed using lmerTest, lsmean, and multcomp packages. All experiments were performed at least three independent times and a representative experimental replicate was chosen for figure, except when indicated otherwise in the legend.

## Data Availability

• RNA-sequencing data was deposited at GEO and are publicly available as of the date of publication. Accession numbers are listed in the key resources table. Microscopy data reported in this paper will be shared by the lead contact upon request. • This paper does not report original code. • Any additional information required to reanalyze the data reported in this paper is available from the lead contact upon request.

## Supplemental information

Document S1. Figures S1–S6

Table S1. Gene Set category for positive regulation of organ growth at 8h, related to Figure 6

Table S2. Gene Set category for lamellipodium assembly at 8h, related to Figure 6

Table S3. Gene Set category for positive regulation of translation at 8h, related to Figure 6

Table S4. Gene Set category for lamellipodium assembly at 16h, related to Figure 6

Table S5. Gene Set category for actin nucleation at 16h, related to Figure 6

Table S6. Genes differently regulated in NP > ctrl (WT) and NP > acs RNAi flies after exposure to *Sm*Db11, related to Figure 6 Results from RNA-sequencing, with the log2FoldChange for all genes that are differently regulated after *SmDb11* oral infection compared to respective sucrose controls for the correspondent time-points.
