# Supplementary material for: Fast drosophila enterocyte regrowth after infection involves a reverse metabolic flux driven by an amino acid transporter
Source: iScience. 2023 Jul 27;26(9):107490. doi: 10.1016/j.isci.2023.107490 (PMC10448536; doi:10.1016/j.isci.2023.107490)
Supplement: Document S1. Figures S1–S6 [file mmc1.pdf]

iScience, Volume *26*

Supplemental information

Fast *drosophila* enterocyte regrowth after infection involves a reverse metabolic flux driven by an amino acid transporter

Catherine Socha, Inês S. Pais, Kwang-Zin Lee, Jiyong Liu, Samuel Liégeois, Matthieu Lestradet, and Dominique Ferrandon

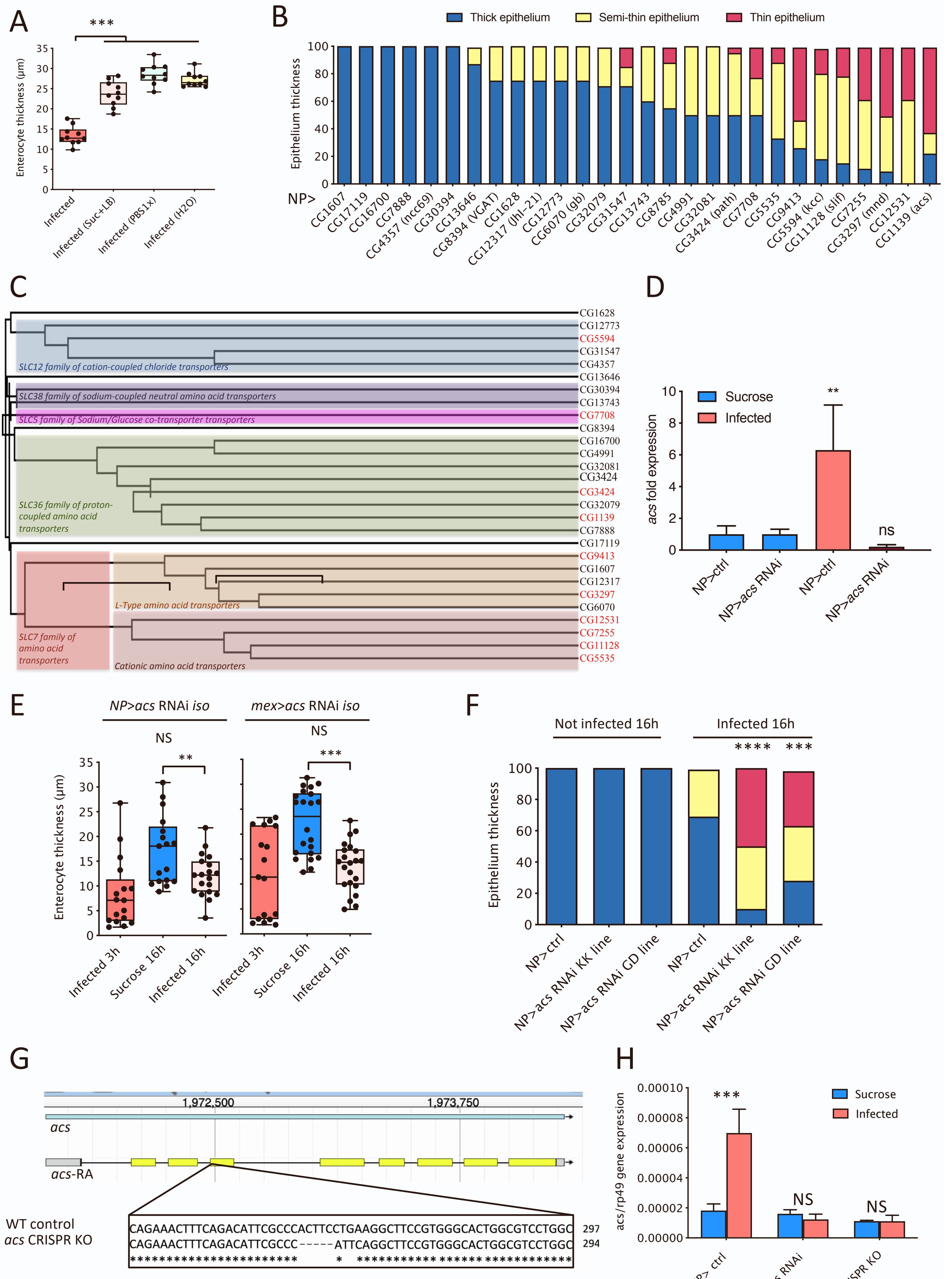

A
B
C
D
E
F
G
H
Enterocyte thickness (µm)
Infected
Infected (Suc+LB)
Infected (PBS1x)
Infected (H2O)
Thick epithelium
Semi-thin epithelium
Thin epithelium
Epithelium thickness
NP>
CG1607
CG17119
CG16700
CG7888
CG4357 (ncc69)
CG30394
CG13646
CG8394 (VGAT)
CG1628
CG12317 (lhl-21)
CG12773
CG6070 (gb)
CG32079
CG31547
CG13743
CG8785
CG4991
CG32081
CG3424 (path)
CG7708
CG5535
CG9413
CG5594 (kcc)
CG11128 (slif)
CG7255
CG3297 (mnd)
CG12531
CG1139 (acs)
SLC12 family of cation-coupled chloride transporters
SLC38 family of sodium-coupled neutral amino acid transporters
SLC5 family of Sodium/Glucose co-transporter transporters
SLC36 family of proton-coupled amino acid transporters
L-Type amino acid transporters
SLC7 family of amino acid transporters
Cationic amino acid transporters
CG5594
CG4357
CG13646
CG8394
CG17119
CG9413
CG3297
CG6070
CG11128
acs fold expression
Sucrose
Infected
NP>ctrl
NP>acs RNAi
ns
NP>acs RNAi iso
mex>acs RNAi iso
NS
Infected 3h
Sucrose 16h
Infected 16h
Not infected 16h
Infected 16h
NP>acs RNAi KK line
NP>acs RNAi GD line
1,972,500
1,973,750
acs
acs-RA
WT control
acs CRISPR KO
CAGAAACTTTCAGACATTCGCCCACTTCCTGAAGGCTTCCGTGGGCACTGGCGTCCTGGC 297
CAGAAACTTTCAGACATTCGCCC-----ATTCAGGCTTCCGTGGGCACTGGCGTCCTGGC 294
acs/rp49 gene expression
NP> ctrl
acs CRISPR KO

Fig. S1

Supplementary Figure 1. Amino acid transporters involved in the fast recovery of the gut epithelium after *Sm*Db11 infection. ***Related to Figures 1&2***

(A) Epithelial thickness quantification of an independent repeat of the experiment displayed in Fig. 1B. (*lm*, *** = p <0.001). **(B)** % epithelium thickness categories were assessed 16h after *Sm*Db11 infection in flies that were knocked down for the different amino acid transporters in the gut enterocytes (with NP> driver). **(C)** Phylogenetic tree based on protein sequence from the different amino acid transporters used to screen in **(B).** Phylogenetic tree generated using UniProt. In red are shown the amino acid transporters that are required to contribute to gut recovery in **(B)**. **(D)** Efficiency of *acs* knockdown in the *NP*> *acs*-RNAi KK line from VDRC. *acs* expression was measured by RT-qPCR on 10 dissected midguts, in triplicates. Expression was determined relative to sucrose controls. *acs* was induced in the midgut during the recovery, 9h post-infection in control but not *NP*> *acs*-RNAi flies (one-way ANOVA, **=p<0.01). **(E)** Control *NP*> *acs* RNAi *iso* and *mex*>*acs* RNAi iso flies were exposed to sucrose or *Sm*Db11 and enterocytes thickness was measured with ImageJ. Each dot represents the average of 10 enterocytes in one midgut. The middle bar of box plots represent the median and the upper and lower limits of the boxes indicate, respectively, the first and third quartile; the whiskers define the minima and maxima (*lmer*, ** = p<0.01; *** = p <0.001, n=7-9 midguts). **(F)** % epithelium thickness categories were assessed 16h after *Sm*Db11 infection or sucrose exposure (non-infected) in *NP*>ctrl, *NP*> *acs*-RNAi KK line and *NP*> *acs*-RNAi GD line. (chi-square, comparing with control, ***=p<0.001,****=p<0.0001) **(G)** Representative map of the gene *acs* with respective exons in yellow, taken from Flybase. CRISPR knock-out mutant for *acs* comprises a deletion of 5bp in the sequence, causing a frame-shift mutation with a non-functional truncated protein. **(H)** *acs* expression level in control flies, *NP*> *acs* RNAi or in the CRISPR KO mutant 9 h post infection. Gene expression was measured by RT-qPCR on 5 dissected midguts, in triplicates. Expression of *acs* increases in control flies but not in *NP*> *acs* RNAi or in the CRISPR KO mutant (*lmer*, *** = p <0.001).
D, H: Bars represent the mean and the error bars display Standard Deviation of the data.

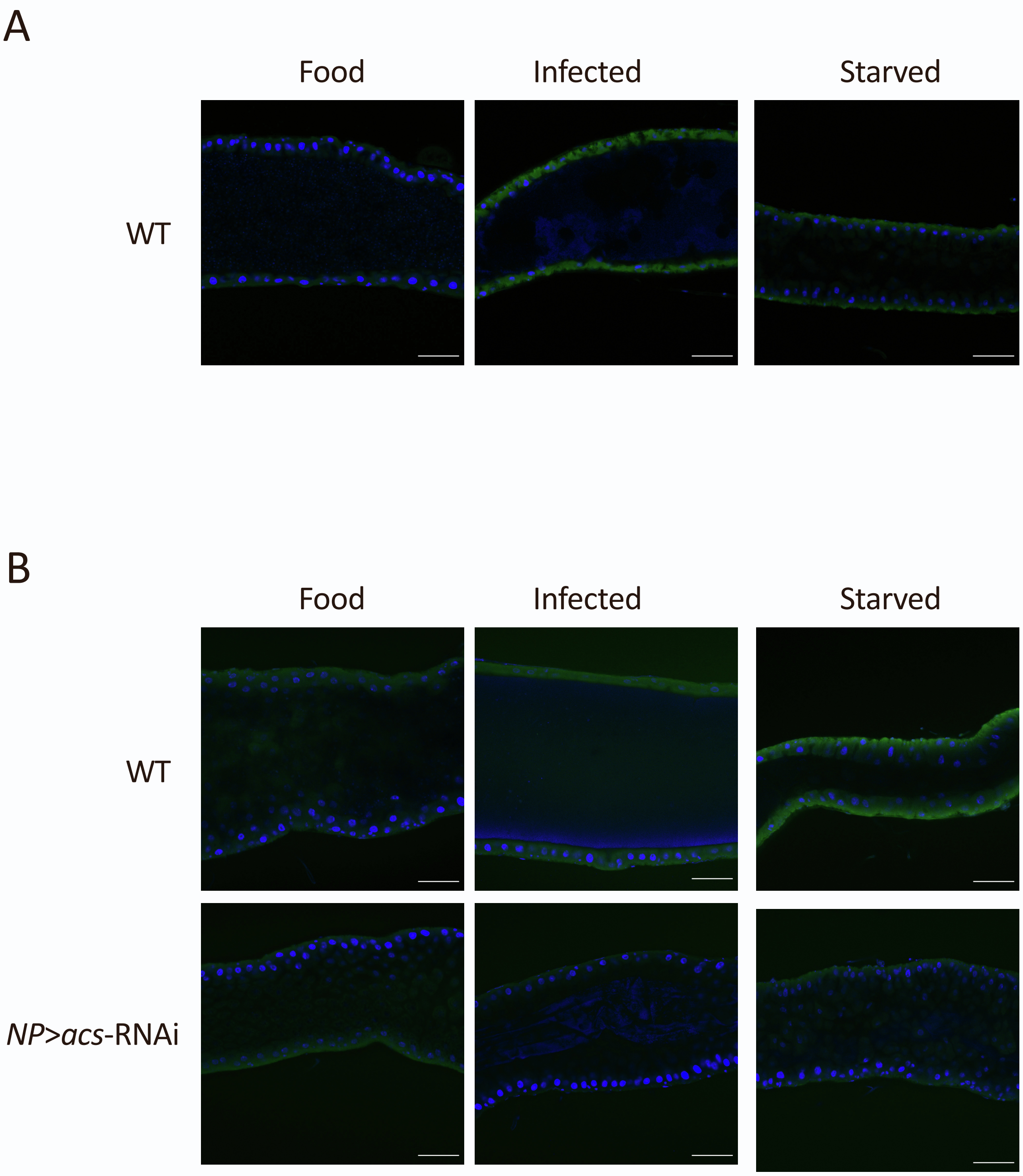
A
Food
Infected
Starved
WT
B
Food
Infected
Starved
WT
NP>acs-RNAi

Fig. S2

Supplementary Figure 2. ***acs*****-dependent uptake in the intestinal epithelium of an injected methionine analogue upon *Sm*Db11 exposure or starvation.** ***Related to Figures 1&4***

(A-B) Flies were injected with 50 µM AHA and exposed to regular food, *Sm*Db11 (infected), sucrose or water (starved). Midguts were dissected 6h later and fluorescence from the incorporated amino-acid was assessed by staining with an alkyne probe. No uptake was observed in the *acs*-silenced midguts (B). Quantification is found respectively in Fig. 1G (A) and Fig. 4C (B). Note that the signal appears to be concentrated in the basal side of enterocytes, where ACS is also located, and possibly other amino-acid transporters, Scale bars are 100 µm.

A

Injection strain: *w[1118]*

Genome Editing:

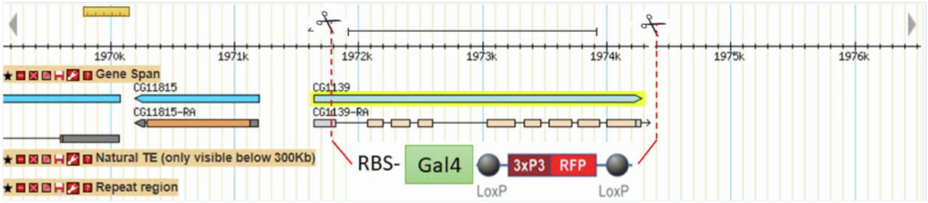

(1) The entire CDS region of *CG1139* will be deleted and replaced by cassette Gal4-RFP.

(2) Cassette Gal4-RFP contains ribosome binding sequence (RBS), Gal4, SV40 polyA terminator, and floxed 3XP3-RFP as a selection marker.

(3) The selection marker 3XP3-RFP contains loxP site, 3X Pax3 promoter, RFP, alpha-Tubulin 3'UTR and loxP site. It facilitates the genetic screening and can be flipped out by Cre recombinase.

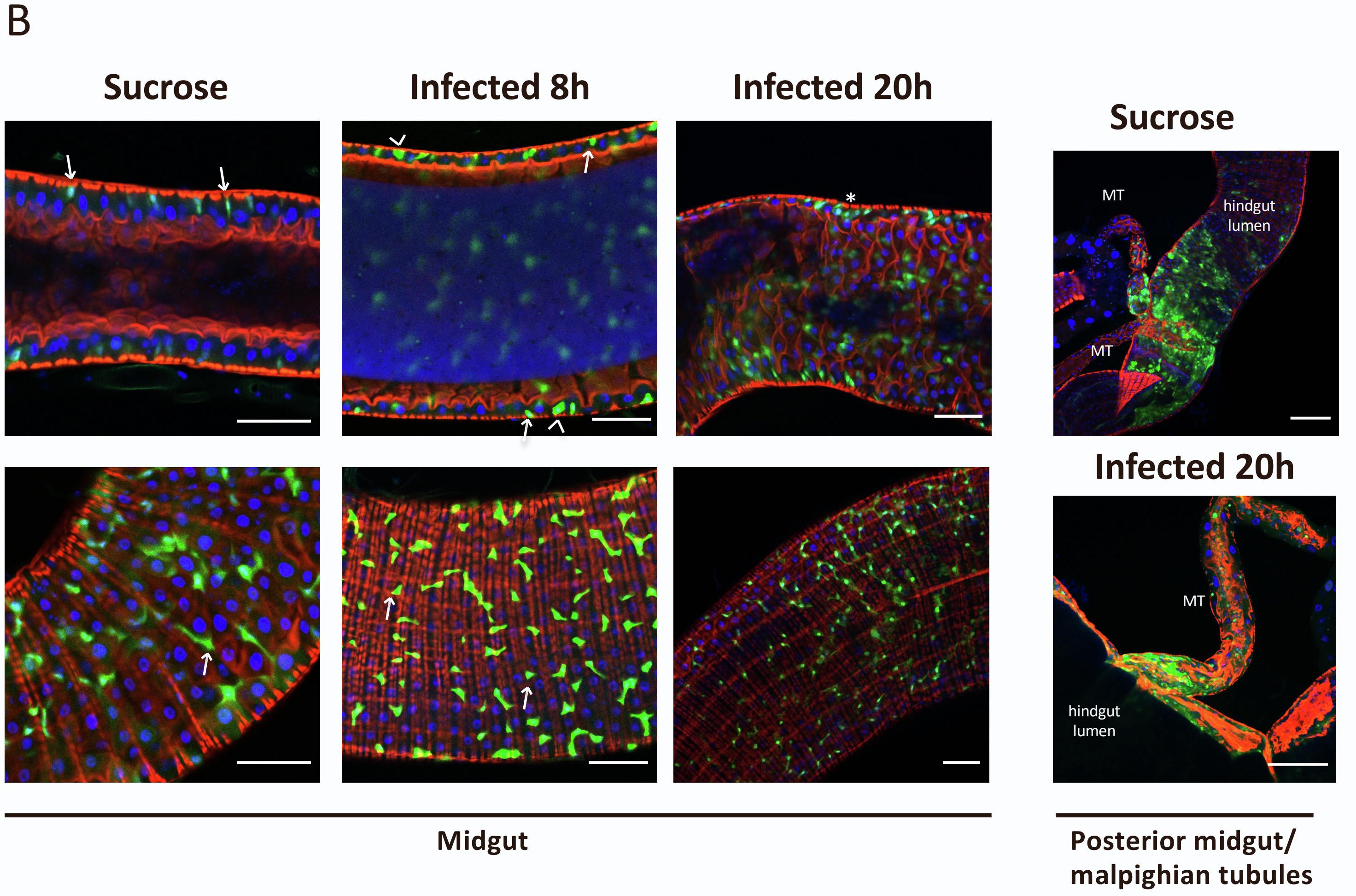

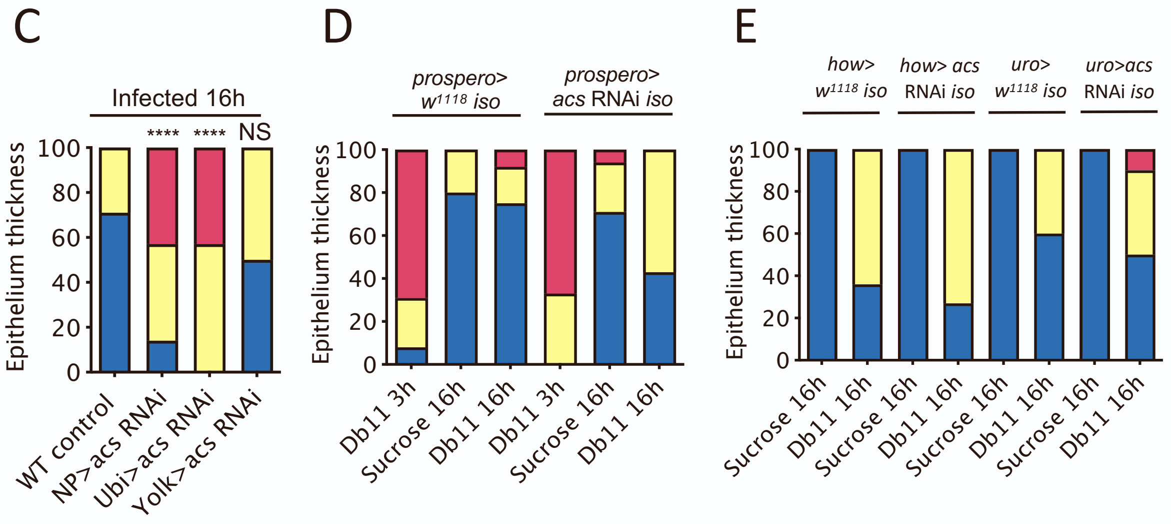

Fig. S3

Supplementary Figure 3. Function of ACS in other host tissues and cell types. ***Related to Figure 3***

(A) Scheme for the Knock-in CRISPR mutant generated for *CG1139/acs* (*acs* KI CRISPR mutant). The entire coding sequence of *CG1139/acs* was deleted and replaced by a Gal4 cassette. **(B)** *acs* KI CRISPR mutant flies were crossed with UAS-GFP flies and the progeny was exposed to sucrose or infected with *Sm*Db11 for 8h and 20h. Cells expressing *acs* are GFP positive cells (green). Blue = DAPI; Red = Actin. Expression of *acs* is present in progenitor cells that resemble ISCs (arrows) and enteroblasts (arrowheads) after sucrose exposure and also after infection (scale for midguts, 50µm). Expression of *acs* is also observed in some enterocytes (star). *acs* is also expressed in Malpighian tubules (MT) and posterior midgut, either in sucrose and infection conditions (scale for posterior midgut and Malpighian tubules, 100µm). n=8-10 guts per condition, 2 independent experimental replicates. **C-E**: Qualitative quantification of intestines in categories according to their epithelial thickness: Thin-red, Semithin-yellow, Thick-blue (n=24-30). **(C)** *acs* was knocked down in enterocytes (*NP>acs* RNAi), whole body (*ubi>acs* RNAi) and in the fat body (*yolk> acs* RNAi). Flies were infected with *Sm*Db11 and the % thickness categories of gut epithelium was assessed 16h post infection. Knocking down *acs* in whole body and in the enterocytes delays the recovery to the thick gut epithelium. (chi-square, comparing with control, ****=p<0.0001) **(D)** *acs* was knocked down in enteroendocrine cells (*prospero>acs* RNAi *iso*). Flies were infected with *Sm*Db11 or exposed to sucrose and % thickness categories of gut epithelium was assessed 3h and 16h post infection. **(E)** *acs* was knocked down in the visceral muscle (*how>acs* RNAi *iso*) and in the Malpighian tubules (*uro>acs* RNAi *iso*). Flies were infected with *Sm*Db11 or exposed to sucrose and the % thickness categories of gut epithelium was assessed 16h post infection.

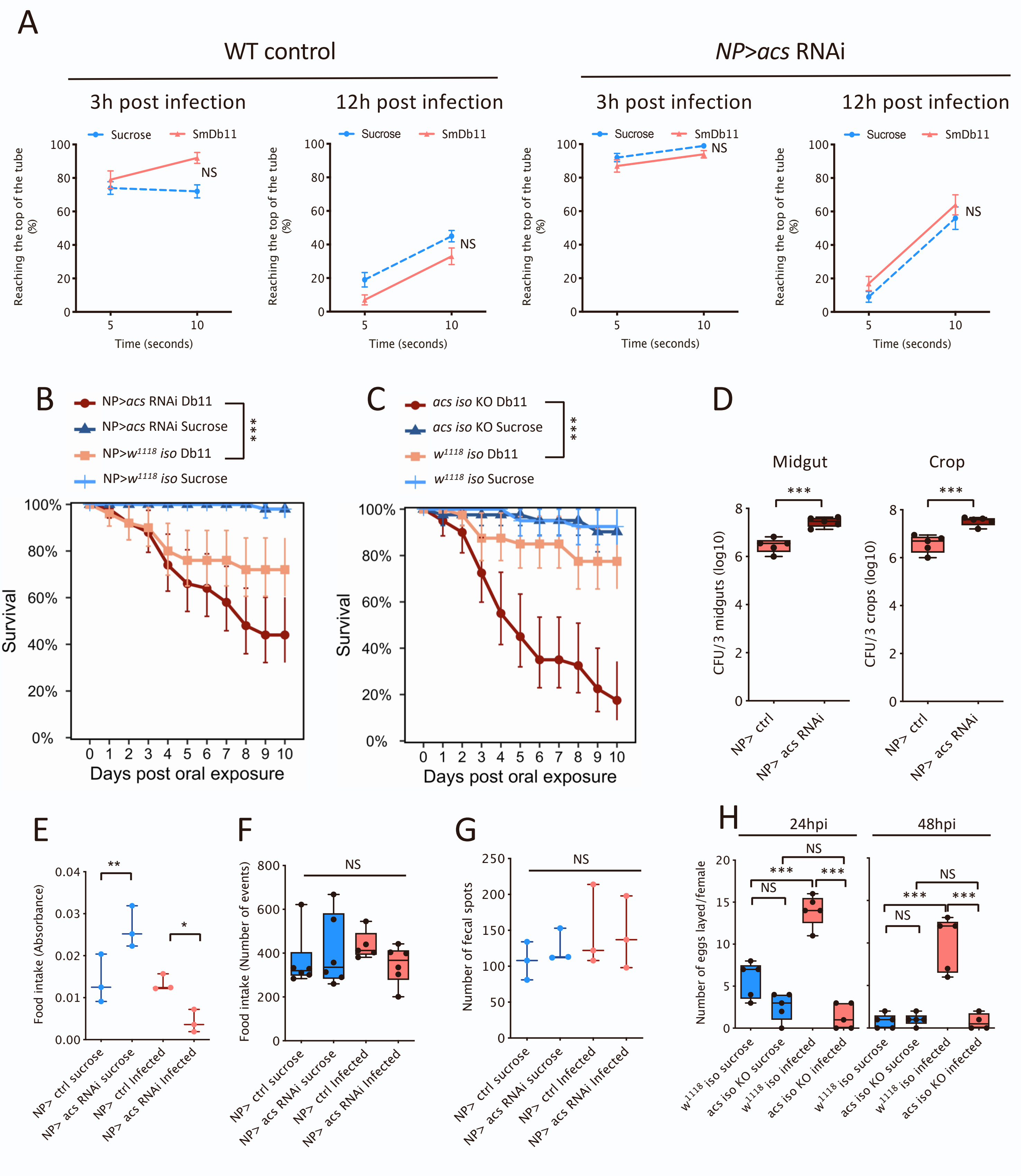

A
WT control
NP>acs RNAi
3h post infection
12h post infection
Sucrose
SmDb11
NS
Reaching the top of the tube (%)
Time (seconds)
B
NP>acs RNAi Db11
NP>acs RNAi Sucrose
NP>w1118 iso Db11
NP>w1118 iso Sucrose
***
Survival
Days post oral exposure
C
acs iso KO Db11
acs iso KO Sucrose
w1118 iso Db11
w1118 iso Sucrose
D
Midgut
Crop
CFU/3 midguts (log10)
CFU/3 crops (log10)
NP> ctrl
NP> acs RNAi
E
Food intake (Absorbance)
NP> ctrl sucrose
NP> acs RNAi sucrose
NP> ctrl Infected
NP> acs RNAi Infected
F
Food intake (Number of events)
G
Number of fecal spots
H
24hpi
48hpi
Number of eggs layed/female
w1118 iso sucrose
acs iso KO sucrose
w1118 iso infected
acs iso KO infected

Fig. S4

Supplementary Figure 4. *acs* is required for increased fitness upon infection. ***Related to Figure 4***

(A) Fly locomotion assessed by Negative Geotaxis Assay in control and *NP>acs* RNAi flies. Flies were either exposed to sucrose or infected with *Sm*Db11 and the assay was performed 3h and 16h post-infection. There are no significant differences between control and *NP>acs* RNAi flies. Error bars display Standard Deviation of the data (one-way ANOVA, n=20, 2 independent experiments). **(B-C)** Survival of *NP>*ctrl and *NP>acs* -RNAi **(B)** or w^{1118} *iso* and *acs iso* CRISPR KO flies **(C)** during chronic infection with *Sm*Db11 OD=1 or exposed to sucrose 50mM. *NP>acs* RNAi and *acs iso* CRISPR KO flies present higher mortality after infection compared to their respective wild-type controls. Error bars display Standard Deviation of the data (*lmer*, ***=*p*-value<0. 001). **(D)** Bacterial loads 16h after *Sm*Db11 infection in *NP>*ctrl or *NP>acs* -RNAi. Each sample (each dot) corresponds to a pool of three midguts or three crops. *NP>acs* RNAi have higher bacterial loads in the midgut and crop compared to control flies (*lmer*, ***=*p*<0.001). **(E)** *NP>* ctrl and *NP>acs* RNAi flies were either infected or exposed to sucrose in a solution containing blue dye for 16h. Flies were collected, and food intake was analyzed by measuring the absorbance of ingested blue dye (*lm*, *=*p*<0.01, **=*p*<0.001, 1 experimental replicate) **(F)** *NP>* ctrl and *NP>acs* RNAi flies were infected or exposed to sucrose using the FLIC system for 16h and number of events was quantified (*lmer*, NS=*p*>0.05) **(G)** *NP>* ctrl and *NP>acs* RNAi flies were either infected or exposed to sucrose in a solution containing blue dye for 16h. Fecal spots were counted on the side of the vials. There are no significant differences between the fly lines (*lm, p*>0.05, n=3 tubes with 10 flies, 1 experimental replicate). **(H)** w^{1118} *iso* and *acs iso* CRISPR KO flies were exposed for 16h to *SmDb11* OD=10. Afterwards, single females with two males were placed in individual vials (n=5 vials) for 24h and transferred to new vials after that period for another 24h. The number of eggs in each vial was counted thus yielding data for egg laying 24h and 48h after moving the flies back to regular fly food (*lmer*, ***=*p*<0.001).

For D, F, H, the middle bar of box plots represents the median and the upper and lower limits of the boxes indicate, respectively, the first and third quartile; the whiskers define the minima and maxima. For E and G, the median bars represent the mean while the error bars display Standard Deviation of the data.

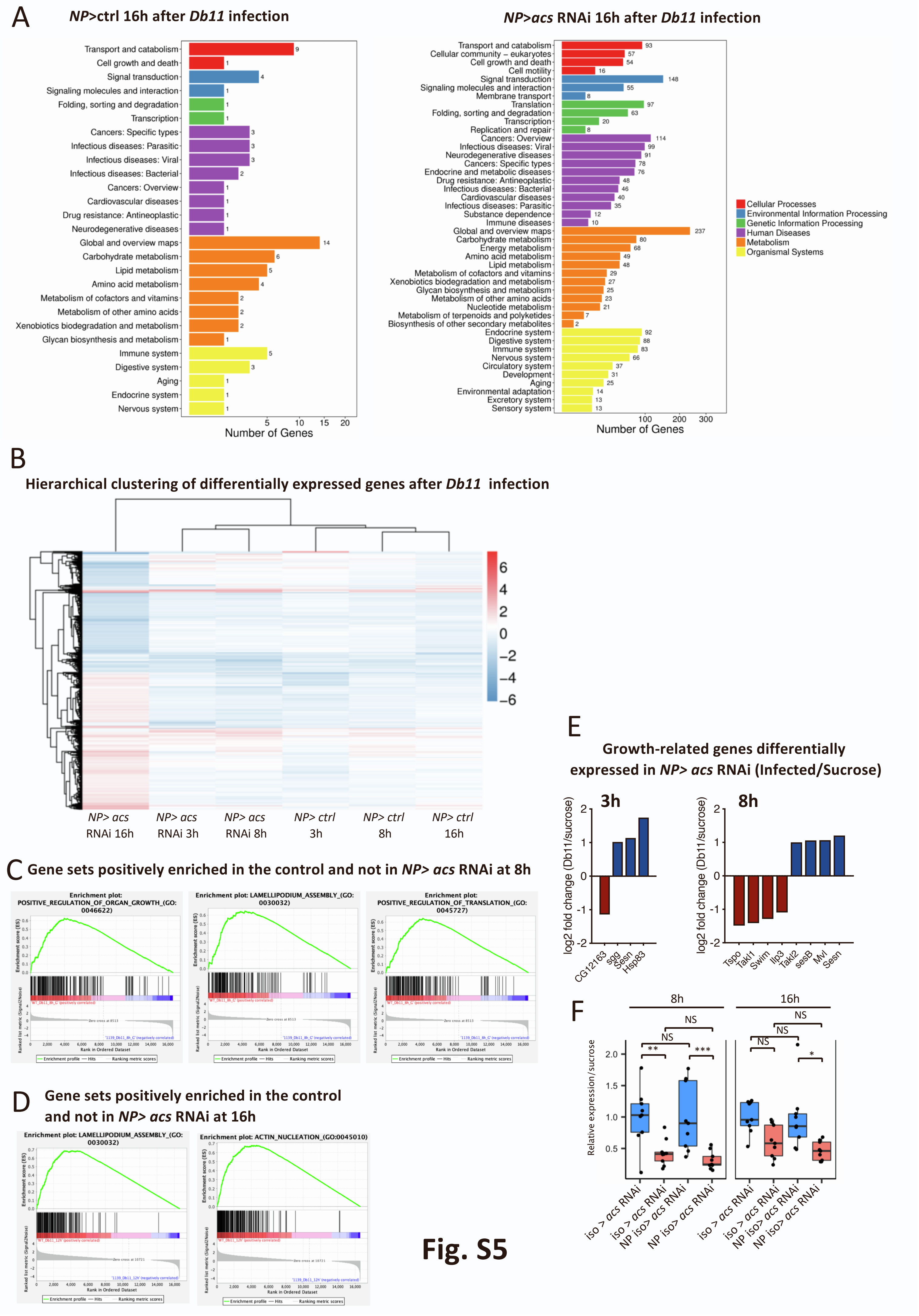
A
NP>ctrl 16h after Db11 infection
Transport and catabolism
Cell growth and death
Signal transduction
Signaling molecules and interaction
Folding, sorting and degradation
Transcription
Cancers: Specific types
Infectious diseases: Parasitic
Infectious diseases: Viral
Infectious diseases: Bacterial
Cancers: Overview
Cardiovascular diseases
Drug resistance: Antineoplastic
Neurodegenerative diseases
Global and overview maps
Carbohydrate metabolism
Lipid metabolism
Amino acid metabolism
Metabolism of cofactors and vitamins
Metabolism of other amino acids
Xenobiotics biodegradation and metabolism
Glycan biosynthesis and metabolism
Immune system
Digestive system
Aging
Endocrine system
Nervous system
Number of Genes
NP>acs RNAi 16h after Db11 infection
Transport and catabolism
Cellular community – eukaryotes
Cell growth and death
Cell motility
Signal transduction
Signaling molecules and interaction
Membrane transport
Translation
Folding, sorting and degradation
Transcription
Replication and repair
Cancers: Overview
Infectious diseases: Viral
Neurodegenerative diseases
Cancers: Specific types
Endocrine and metabolic diseases
Drug resistance: Antineoplastic
Infectious diseases: Bacterial
Cardiovascular diseases
Infectious diseases: Parasitic
Substance dependence
Immune diseases
Global and overview maps
Carbohydrate metabolism
Energy metabolism
Amino acid metabolism
Lipid metabolism
Metabolism of cofactors and vitamins
Xenobiotics biodegradation and metabolism
Glycan biosynthesis and metabolism
Metabolism of other amino acids
Nucleotide metabolism
Metabolism of terpenoids and polyketides
Biosynthesis of other secondary metabolites
Endocrine system
Digestive system
Immune system
Nervous system
Circulatory system
Development
Aging
Environmental adaptation
Excretory system
Sensory system
Number of Genes
Cellular Processes
Environmental Information Processing
Genetic Information Processing
Human Diseases
Metabolism
Organismal Systems
B
Hierarchical clustering of differentially expressed genes after Db11 infection
NP> acs RNAi 16h
NP> acs RNAi 3h
NP> acs RNAi 8h
NP> ctrl 3h
NP> ctrl 8h
NP> ctrl 16h
C Gene sets positively enriched in the control and not in NP> acs RNAi at 8h
Enrichment plot: POSITIVE_REGULATION_OF_ORGAN_GROWTH_(GO: 0046622)
Enrichment plot: LAMELLIPODIUM_ASSEMBLY_(GO: 0030032)
Enrichment plot: POSITIVE_REGULATION_OF_TRANSLATION_(GO: 0045727)
D Gene sets positively enriched in the control and not in NP> acs RNAi at 16h
Enrichment plot: LAMELLIPODIUM_ASSEMBLY_(GO: 0030032)
Enrichment plot: ACTIN_NUCLEATION_(GO:0045010)
E
Growth-related genes differentially expressed in NP> acs RNAi (Infected/Sucrose)
3h
8h
log2 fold change (Db11/sucrose)
CG12163
sgg
Sesn
Hsp83
Tspo
Tak1
Swim
Ilp3
Takl2
sesB
Mvl
Sesn
F
8h
16h
Relative expression/sucrose
iso > acs RNAi
iso > acs RNAi
NP iso> acs RNAi
NP iso> acs RNAi
NS
**
***
*

Fig. S5

Supplementary Figure 5. Genes differentially expressed between NP>*acs* RNAi and control flies after infection. ***Related to figure 6***

(A) KEGG pathway enrichment analysis for the RNA sequencing performed on guts from *NP*>ctrl and *NP*>*acs*-RNAi flies, at 16h post *Db11* infection compared with respective sucrose controls. The fact that the *NP*>ctrl samples were generally more spread out than the *NP*>*acs* RNAi samples, especially those corresponding to wild-type flies fed on sucrose (Fig. 6A) may limit the power of the KEGG pathway analysis in *NP*>ctrl flies, as reflected by the low number of genes revealed in this panel. **(B)** Hierarchical clustering of genes differently expressed at 3h, 8h and 16h post *Sm*Db11 infection, relative to the correspondent sucrose control. Red correspond to up-regulated genes and blue to down-regulated genes. **(C, D)** Gene set enrichment analysis was performed on the RNA sequencing results to compare enriched gene sets between *NP*> ctrl and *NP*>*acs* RNAi. **(C)** Genes involved in positive regulation of growth, lamellipodium formation and positive regulation of translation are positively enriched at 8h post infection in *NP*> ctrl and not in *NP*>*acs* RNAi; see also Supplementary tables 1-3. **(D)** At 16h post infection, there is an enrichment of genes involved in lamellipodium formation, actin nucleation and negative regulation of growth in the *NP*> ctrl and not in *NP*>*acs* RNAi (see also Supplementary tables 4-5). **(E)** Genes involved in growth that are up- or down-regulated in *acs* RNAi 3h and 8h after infection (relative to sucrose). **(F)** Expression of *Myc* was measured by RT-qPCR in dissected midguts of *NPiso*>*acs* RNAi and w^{1118} *iso*>*acs* RNAi flies 8h and 16h post infection. *Myc* steady-state transcripts are down-regulated at 8h in both flies but at 16h are only down-regulated in *NPiso*>*acs* RNAi and not significantly different in control flies. T he middle bar of box plots represent the median and the upper and lower limits of the boxes indicate, respectively, the first and third quartile; the whiskers define the minima and maxima (*lmer*, *=p<0.05, **=p<0.01, ***=p<0.001, data shown is pooled from 3 independent experiments); blue: flies fed on sucrose, red: flies fed on *Sm*Db11 solution.

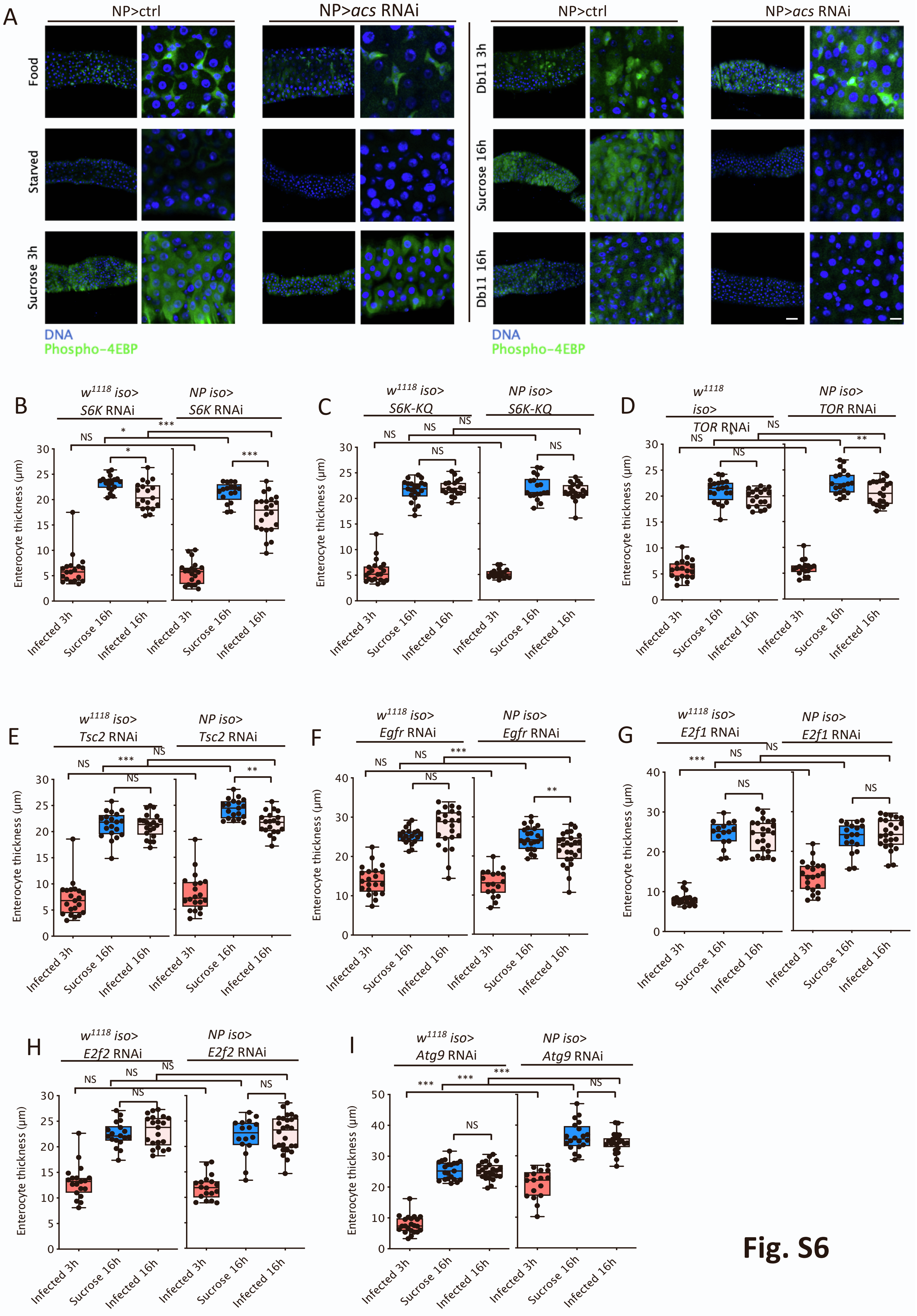
A
NP>ctrl
NP>acs RNAi
Food
Starved
Sucrose 3h
Db11 3h
Sucrose 16h
Db11 16h
DNA
Phospho-4EBP
B
w1118 iso> S6K RNAi
NP iso> S6K RNAi
C
w1118 iso> S6K-KQ
NP iso> S6K-KQ
D
w1118 iso> TOR RNAi
NP iso> TOR RNAi
E
w1118 iso> Tsc2 RNAi
NP iso> Tsc2 RNAi
F
w1118 iso> Egfr RNAi
NP iso> Egfr RNAi
G
w1118 iso> E2f1 RNAi
NP iso> E2f1 RNAi
H
w1118 iso> E2f2 RNAi
NP iso> E2f2 RNAi
I
w1118 iso> Atg9 RNAi
NP iso> Atg9 RNAi
Enterocyte thickness (μm)
Infected 3h
Sucrose 16h
Infected 16h
NS
*
**
***

Fig. S6

Supplementary Figure 6. TOR pathway activity decreases in *NP>acs*-RNAi flies but is not strongly involved in recovery of the epithelium after infection. ***Related to figure 6***

(A) Confocal pictures of dissected midguts. Blue=DNA; Green=phospho-4EBP (P-4EBP). Midguts were stained with the P-4EBP antibody to detect when and where the TOR pathway was active. P-4EBP signal decreased 16h post-infection in *acs* knock-down flies, as well as when fed sucrose for 16h. n=20, 2 independent experiments. Scale bars are respectively 50 µm (low magnification panels) and 14 µm (high magnification panels) **(B, D-I)** Several genes were knocked down with RNAi using the enterocyte specific driver NP*iso* and w^{1118} *iso* as a control. A dominant negative form of S6K (S6K-KQ) was also used in **(C)**. Females were exposed to sucrose or infected with *Sm*Db11 and enterocyte thickness was measured at the shown time-points. Some of these genes when knocked down with NP*iso* driver presented a significative difference in the enterocyte thickness 16h post infection when compared to their sucrose controls (**D**, **E**, **F**, *lmer* ** = $p < 0.01$). However, this difference was never higher than 2 µm. Only the silencing of S6K showed a more significant lack of recovery **(B)** (*lmer*, *** = $p<0.001$), but this was not observed with the dominant negative form (**C**). B-I: each dot corresponds to 1 midgut, data shown corresponds to a pool of 2 independent replicates. The middle bar of box plots represent the median and the upper and lower limits of the boxes indicate, respectively, the first and third quartile; the whiskers define the minima and maxima.
